# CD8 T cells targeting adapted epitopes in chronic HIV infection promote dendritic cell maturation and CD4 T cell *trans*-infection

**DOI:** 10.1371/journal.ppat.1007970

**Published:** 2019-08-09

**Authors:** Kai Qin, Sushma Boppana, Victor Y. Du, Jonathan M. Carlson, Ling Yue, Dario A. Dilernia, Eric Hunter, Robbie B. Mailliard, Simon A. Mallal, Anju Bansal, Paul A. Goepfert

**Affiliations:** 1 Department of Medicine, University of Alabama at Birmingham, Birmingham, Alabama, United States of America; 2 The Salk Institute for Biological Studies, La Jolla, California, United States of America; 3 Microsoft Research, Redmond, Washington, United States of America; 4 Emory Vaccine Center at Yerkes National Primate Research Center and Department of Pathology and Laboratory Medicine, Emory University, Atlanta, Georgia, United States of America; 5 Department of Infectious Diseases and Microbiology, University of Pittsburgh, Pittsburgh, Pennsylvania, United States of America; 6 Division of Infectious Diseases, Department of Medicine, Vanderbilt University Medical Center, Nashville, Tennessee, United States of America; Vaccine Research Center, UNITED STATES

## Abstract

HIV-1 frequently escapes from CD8 T cell responses via HLA-I restricted adaptation, leading to the accumulation of adapted epitopes (AE). We previously demonstrated that AE compromise CD8 T cell responses during acute infection and are associated with poor clinical outcomes. Here, we examined the impact of AE on CD8 T cell responses and their biological relevance in chronic HIV infection (CHI). In contrast to acute infection, the majority of AE are immunogenic in CHI. Longitudinal analyses from acute to CHI showed an increased frequency and magnitude of AE-specific IFNγ responses compared to NAE-specific ones. These AE-specific CD8 T cells also were more cytotoxic to CD4 T cells. In addition, AE-specific CD8 T cells expressed lower levels of PD1 and CD57, as well as higher levels of CD28, suggesting a more activated and less exhausted phenotype. During CHI, viral sequencing identified AE-encoding strains as the dominant quasispecies. Despite increased CD4 T cell cytotoxicity, CD8 T cells responding to AE promoted dendritic cell (DC) maturation and CD4 T cell *trans*-infection perhaps explaining why AE are predominant in CHI. Taken together, our data suggests that the emergence of AE-specific CD8 T cell responses in CHI confers a selective advantage to the virus by promoting DC-mediated CD4 T cell *trans*-infection.

## Author summary

HIV-1 infection remains a critical public health threat across the world. Over the past two decades, CD8 T cells have been clearly shown to exert immune pressure on HIV and drive viral adaptation. Previously, our group reported that such HLA-I associated adaptations can predict clinical outcomes and are beneficial to HIV-1 as CD8 T cells are unable to recognize epitopes with adaptation in acute HIV infection. However, it is still unclear how HIV-1 adaptation impacts CD8 T cells during chronic HIV infection. In this study, we observed an enhancement of CD8 T cell responses targeting adapted epitopes in chronic infection. Although these responses were cytotoxic, they also exhibited a “helper” effect by promoting viral infection of CD4 T cells via interaction with dendritic cells. This phenomenon may contribute to the persistence of adapted viruses. In summary, these findings present a novel mechanism of CD8 T cell driven HIV-1 adaptation.

## Introduction

During natural SIV/HIV infection, CD8 T cells have been shown to be important in viral control [1–6]. CD8 T cells were first shown to play a critical role in maintaining viral suppression in the SIV/non-human primate (NHP) model [1–3]. In the hyper-acute phase of HIV-1 infection, the kinetics of CD8 T cell activation, as well as the magnitude of response, directly correlated with a lower viral load set point [4]. Given that CD8 T cells play an important role in viral control during natural HIV-1 infection, a deeper understanding of how CD8 T cell function is influenced by the virus could greatly inform the development of optimal vaccination and functional cure strategies.

One major obstacle to inducing effective CD8 T cell responses is the rapid rate of viral mutation, promoting the selection of escape mutations which in turn increase viral fitness by diminishing the cytotoxic CD8 T cell response [7–10]. Our group and others have used population level analyses to define HIV-1 adaptation through HLA-I associated polymorphisms [11–14]. We use the term adaptation rather than escape to be more inclusive of mutations that afford a benefit to the virus but that do not necessarily result in decreased immune recognition. We have termed epitopes containing these HLA-I associated polymorphisms as adapted epitopes (AE), and those epitopes lacking any evidence of HLA-I associated changes as non-adapted epitopes (NAE). Using this approach, we previously demonstrated that individuals infected with a transmitted founder virus (TFV), highly adapted to their HLA-I alleles, were found to have accelerated CD4 T cell decline and an increased viral load (VL) set point [14]. Furthermore, during acute infection, we found AE were poorly immunogenic, suggesting that HIV-1 adaptation is primarily associated with early escape from CD8 T cell responses [14]. However, it remains unclear how AE affect CD8 T cell responses in chronic HIV infection (CHI).

Not all adapted epitopes result in complete (classical) escape from the CD8 T cell response. In fact, in spite of decreased recognition in acute infection, the overall predicted HLA-I binding affinity is comparable for many non-adapted and adapted epitopes, suggesting that AE can be presented to CD8 T cells [14]. And, it was previously shown that *de novo* CD8 T cell responses can be generated in response to emerging escape mutations in chronic HIV-1 infection [15]. Our group and others have previously demonstrated that CD8 T cell cross-reactivity broadens from acute to chronic infection [16–18]. Interestingly, there has also been increasing evidence that HIV-1 adaptations may confer several viral benefits other than just classical escape, such as increasing viral fitness [19, 20], compensating for fitness costly mutations [21, 22], and acting as a “decoy” by drawing CD8 T cell responses away from other epitopes [23]. Another intriguing viral advantage non-classical adaptation was put forth by *Mailliard et al*. where some variant epitopes elicited a “helper-like” CD8 T cell phenotype, which contributed to viral *trans*-infection by promoting monocyte derived DC maturation and inducing a pro-inflammatory response [24]. Along the same lines, another recent study showed that resistance of monocyte derived macrophages to HIV-specific cytotoxic CD8 T cell killing promoted inflammation whereas CD8 T cells rapidly killed CD4 T cell targets, suggesting that CD8 effectors may yield different outcomes depending on the type of target cell [25].

Our present study aimed to assess the immunogenicity of AE in chronic HIV-1 infection and to determine how these HLA-I restricted adaptations might alter the host immune response and benefit the virus. We found that contrary to acute infection, CD8 T cells increasingly target AE with higher *in vitro* cytotoxicity in chronic infection. In spite of this apparent increase in immune pressure, AE remained the dominant epitope encoded by viral quasispecies in chronic infection. We further found that AE-specific CD8 T cells promoted viral *trans*-infection from mature DCs to CD4 T cells, suggesting a mechanism by which HIV-1 adaptation confers a viral advantage other than direct immune evasion.

## Results

### Adapted epitope-specific CD8 T cell IFNγ responses are enriched in chronic HIV-1 infection

**Fig 1:**
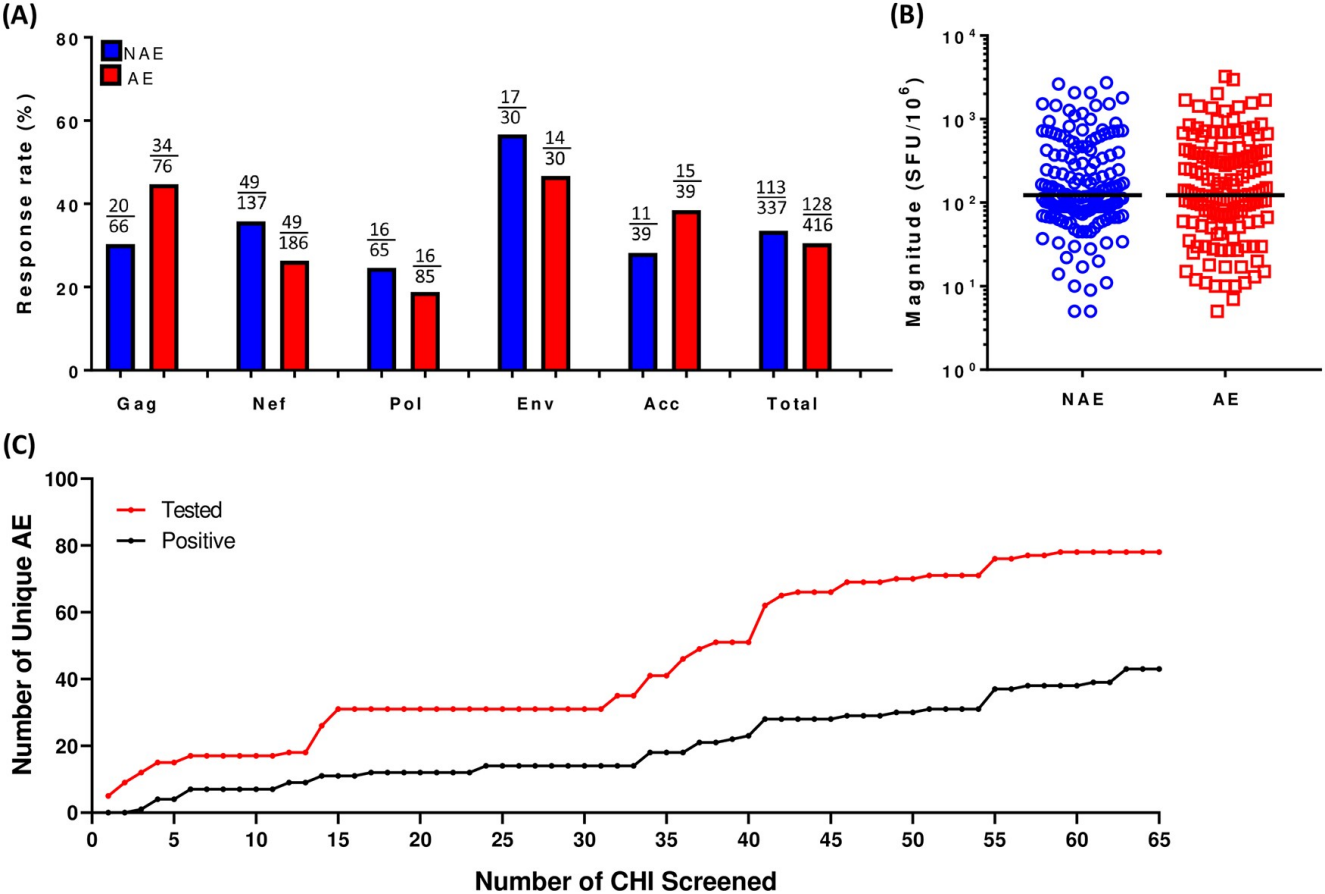
AE specific CD8 IFNγ responses are similar to the NAE ones in chronic infection. CD8 T cell response to NAE and AE obtained from HIV-1 chronically-infected patients (N = 65) is assessed using an IFNγ ELISpot assay. **(A)** Number of immunogenic epitopes/number of epitopes tested (stratified by each protein and in total) is indicated as a response rate. **(B)** Overall magnitude of response to NAE and AE is shown. Each dot represents a single NAE or AE response. Only NAE/AE pairs with at least one positive response are compared. **(C)** The percentage of AE tested that elicit a positive response in at least one individual against the total number of individuals screened for responses. Fisher’s exact test for (**A**) and mixed effects model for (**B)** were used to determine statistical significance.

In order to determine the immunogenicity of AE in chronic infection, we assayed PBMC samples from 65 HIV-1 chronically infected patients (Table 1). Each sample was tested for IFNγ-producing CD8 T cell responses for predicted NAE and AE pairs that were restricted by their HLA-I alleles using an ELISpot assay. Contrary to what we have seen in acute infection [14], the frequency and magnitude of responses towards NAE and AE were similar in chronic infection regardless of protein specificity (Fig 1A and 1B). We also compared the CD8 T-cell responses restricted by protective HLA alleles (B*27, B*57, and B*5801) with CD8 responses restricted by neutral alleles (all other HLA-I alleles), and did not see any significant differences between these 2 allelic groups.

**Table 1:** Demographics and clinical parameters of chronic clade B HIV-1 infected individuals.

CHI	HLA-I Alleles						Gender^a^	VL^b^	CD4^c^	Race^d^	ART^e^
	A1	A2	B1	B2	C1	C2					
1	0101	0301	0702	0809	0701	0702	M	3.4	687	C	Yes
2	0201	0301	0702	1510	0701	0702	M	4.3	132	AA	Yes
3	0201	0301	0702	4001	0304	0702	M	4.4	405	C	Yes
4	0301	7401	0702	1503	0202	0702	M	3.5	562	AA	Yes
5	0201	1101	3501	5101	0401	1402	M	4.4	656	C	No
6	0301	0301	0702	4402	0702	04	M	4.0	531	C	No
7	0201	3201	1501	3501	0202	0304	M	4.1	153	C	Yes
8	0201	0301	3501	4402	0401	0501	M	5.0	964	C	No
9	0201	3101	2705	4402	0202	0501	M	5.6	586	C	Yes
10	0301	2301	4101	5301	0401	0802	M	3.4	579	AA	No
11	3201	6802	3501	5301	0401	0401	M	3.5	659	AA	Yes
12	1101	3201	2705	4001	0102	0304	M	2.8	991	C	No
13	0201	2402	2705	4002	0202	0202	M	1.7	495	C	Yes
14	0201	0201	4402	5701	0501	0602	M	3.5	712	C	No
15	2301	3303	0801	5301	0304	0602	M	2.6	9	AA	Yes
16	0101	0101	0801	2705	0701	1502	M	4.2	848	C	No
17	0101	0301	0801	4402	0501	0701	M	2.4	122	C	Yes
18	2501	3002	0702	3901	1203	1502	F	4.9	890	AA	Yes
19	0301	7401	3501	4403	0401	1601	F	2.5	490	AA	Yes
20	1101	2301	1402	3701	0602	0802	F	3.6	521	AA	Yes
21	0101	0301	0702	0801	0701	0702	M	3.9	719	C	Yes
22	2301	3303	0702	1510	0304	0702	M	3.4	281	AA	Yes
23	0201	2601	3501	3501	0401	0401	M	1.7	296	AA	Yes
24	0201	0301	0702	0702	0702	0702	M	3.0	311	C	Yes
25	0201	0205	4402	5801	0501	0701	F	4.1	713	C	No
26	0201	0301	0702	0702	0702	0702	M	3.9	564	C	No
27	2402	6802	3501	4501	0401	0602	M	1.7	496	AA	Yes
28	0201	0301	0702	5701	0602	0702	F	3.3	492	C	No
29	0201	0201	0702	4402	0304	0702	F	4.7	384	C	Yes
30	3002	3201	0702	1401	0702	0802	F	2.9	824	AA	No
31	0201	0301	1501	4402	0304	0501	M	3.9	654	C	Yes
32	3002	6601	1503	3501	0210	0401	M	3.6	487	AA	Yes
33	2902	6802	0702	5801	0701	0702	M	3.3	331	AA	Yes
34	1101	2301	1402	3501	0401	0802	M	5.0	224	AA	Yes
35	2301	6801	3501	5301	0401	0401	F	1.7	668	AA	NA^f^
36	2301	74	4403	5701	0401	18	M	4.6	623	AA	NA
37	3004	6802	4403	5701	0701	0701	F	1.7	1150	C	NA
38	23	74	1503	3501	0202	04	F	4.1	317	AA	NA
39	23	34	15	53	04	05	M	3.6	241	AA	Yes
40	2301	2301	0702	5301	0401	0702	F	3.2	649	AA	Yes
41	0301	1101	0702	3501	0701	0702	F	3.8	568	AA	NA
42	23	33	07	53	06	15	F	4.0	405	AA	NA
43	2301	3002	5701	8101	0701	0802	F	1.7	628	AA	NA
44	2301	3002	2705	5301	0202	0401	F	NA	112	AA	NA
45	2301	6802	1401	5301	0401	0802	F	4.1	316	AA	NA
46	23	3601	0702	3501	07	07	F	5.7	4	AA	NA
47	23	01	44	53	04	06	F	3.2	654	AA	Yes
48	0101	0201	4402	5703	05	0602	M	4.0	459	AA	NA
49	33	74	44	53	04	04	F	4.3	392	AA	Yes
50	0202	0202	44	53	04	04	F	1.9	383	NA	Yes
51	33	34	44	53	04	04	F	4.7	NA	AA	Yes
52	0301	6601	3501	4102	0401	1701	F	4.3	532	AA	NA
53	3301	6801	3501	5101	0401	0401	F	1.7	1142	AA	NA
54	0101	0201	3501	8101	1601	1801	F	3.9	477	AA	NA
55	3201	3601	3501	1401	0401	0802	F	3.5	514	AA	NA
56	2402	2402	3501	3517	0401	0401	F	4.1	586	AA	NA
57	02	30	3501	1302	04	06	F	3.6	418	AA	NA
58	01	03	3501	5201	04	NA	F	3.0	262	AA	Yes
59	2402	2402	3501	3527	0102	0401	F	NA	774	C	NA
60	0202	0207	3501	1402	04	08	F	1.7	1175	AA	Yes
61	03	66	3501	1510	04	04	F	3.2	188	AA	NA
62	02	29	3501	5101	04	15	F	4.2	NA	AA	Yes
63	02	3001	3501	4201	0702	1701	F	NA	490	AA	NA
64	6801	6802	35	15	03	04	F	5.3	66	AA	NA
65	30	33	07	35	04	15	F	3.4	677	AA	NA

^a^ M: Male, F: Female;

^b^ Log Plasma HIV-1 RNA (copies/ml);

^c^ Absolute CD4 T cell counts (cells/ml);

^d^ C: Caucasian, AA: African American;

^e^ Active antiretroviral therapy status;

^f^ NA: not available.

More than half of AE (56.76%) were immunogenic in chronic infection; however, it is clear that the more often an AE was tested, the higher the chance we saw a response in at least one individual (Fig 1C). In fact, when we looked at unique AE that were tested in at least five different individuals, we found that 80% of these AE elicited CD8 T cell IFN-γ response in at least one patient. Interestingly, only 12.7% (14/110) of the adaptations studied significantly impaired the predicted HLA binding changing the epitopes from strong to weak binder or from weak to no binder as defined by the NetMHC (S1 Table). Additionally, only 33.6% (37/110) of adaptations were located at anchor positions, defined here as the P2 or C-terminal residue. Taken together, our current results indicate that a significant proportion of adapted epitopes are immunogenic in chronic infection, suggesting these mutations are non-classical adaptation.

**Fig 2:**
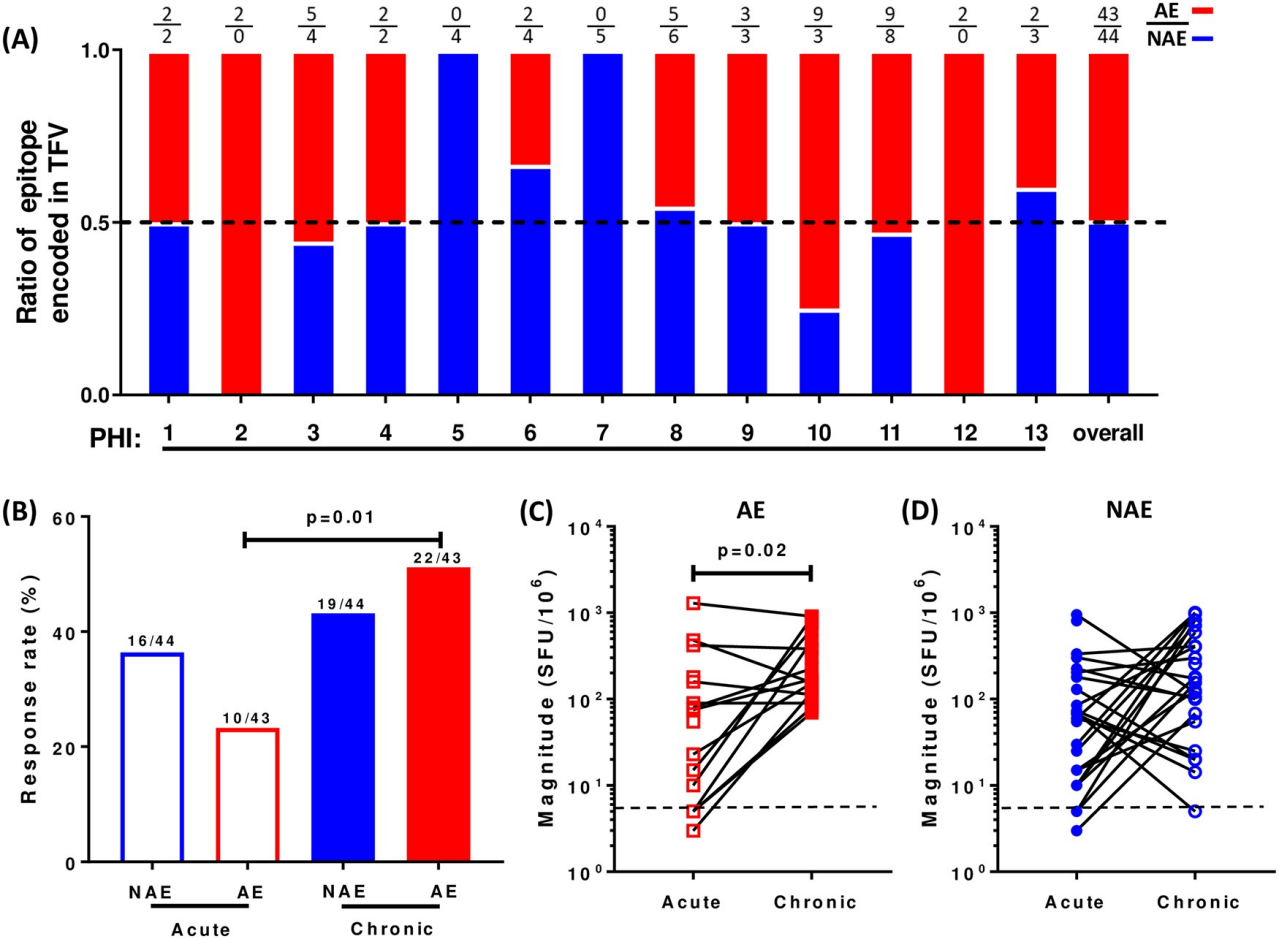
AE specific CD8 IFNγ responses are enriched from acute to chronic infection. **(A)** NAE and AE ratio in TFV sequence for each individual in acute infection was shown. The overall NAE and AE ratio was calculated by dividing the sum of all NAE or AE by the sum of all epitopes. Dotted line is an epitope ratio of 0.5. (**B-D**) Longitudinal analysis of TFV encoding epitope specific CD8 T cell IFNγ responses for each individual (N = 13) during acute (mean DPI = 37±19) and chronic infection (mean DPI = 511±529). **(B)** Response rate to NAE and AE during acute and chronic infection is shown. **(C)** AE and **(D)** NAE response magnitude during acute and chronic infection. Dotted lines represent the cut-off criteria for a positive response. Fisher’s exact 2x2 test was used in (**B**) and Wilcoxon matched-pairs signed rank test was used in (**C and D)** to determine statistical significance.

Next, we evaluated the development of these responses longitudinally. We tested transmitted NAE and AE encoded by the TFV in 13 individuals (Table 2) sampled at both acute and chronic infection time points for IFNγ responses by ELISpot assay. TFV encoded HIV pre-adaptation to CD8 T cells was very common. Overall, 36 unique NAE and 37 unique AE were tested in these 13 individuals, the majority (61/73) of which were tested in one patient and the others in two or three patients (S2 Table). Although some TFVs were enriched with either NAE or AE, we observed overall half of HLA restricted epitopes in TFVs were pre-adapted in this cohort (S2 Table, Fig 2A). The AE-specific CD8 T cells demonstrated a higher response rate (p = 0.01) and magnitude (p = 0.02) during chronic infection compared to acute infection, while the NAE-specific CD8 T cell responses remained similar (Fig 2B, 2C and 2D). Collectively, these data indicate that CD8 T cell IFNγ responses targeting transmitted AE increase significantly in frequency and magnitude from acute into chronic infection.

**Table 2:** Demographics and clinical parameters of acute clade B HIV-1 infected individuals followed longitudinally into chronic infection.

PHI	HLA-I Alleles						Gender^a^	Race^b^	ART start^c^	Acute infection				Chronic infection		
	A1	A2	B1	B2	C1	C2				Fiebig^d^	DPI^e^	VL^f^	CD4^g^	DPI	VL	CD4
1	03:01	66:01	42:01	58:02	06:02	17:01	M	AA	37	I	42	4.7	932	181	5.0	726
2	02:01	02:05	35:01	44:03	04:01	04:01	M	AA	33	I	33	7.1	230	923	5.1	358
3	02:01	24:01	07:02	07:02	07:02	07:02	M	C	23	I	23	7.5	237	284	4.3	806
4	11:01	11:01	35:01	51:01	04:01	15:02	M	C	58	I	30	5.2	286	75	1.9	599
5	68:01	68:01	15:03	58:02	02:10	06:02	M	AA	29	I	16	6.5	719	299	5.9	430
6	02:01	03:01	07:02	14:02	07:02	08:02	M	C	67	II	21	6.0	24	824	4.1	478
7	03:01	23:01	07:02	44:03	04:01	07:02	M	C	28	II	31	5.5	415	106	2.8	359
8	02:01	03:01	07:02	07:02	07:02	07:02	M	C	27	II	31	4.3	479	886	1.7	842
9	23:01	36:01	44:02	44:03	03:02	04:01	M	AA	68	VI	61	4.5	509	278	2.9	638
10	01:01	02:01	08:01	44:02	07:01	07:04	M	C	47	V	45	5.0	389	312	5.0	571
11	30:02	34:02	07:02	35:01	04:01	07:02	M	AA	69	III	34	5.3	761	237	1.7	976
12	01:01	24:02	55:01	55:01	03:03	03:03	M	C	93	I	30	5.0	717	260	1.7	1301
13	01:01	23:01	08:01	52:01	07:01	12:02	M	C	408	VI	90	5.7	575	1979	1.7	979

^a^ M: Male, F: Female;

^b^ C: Caucasian, AA: African American;

^c^ ART start date (number of days between estimated date of infection and date of first ART);

^d^ Fiebig stage at which single genome amplification (SGA) was done to resolve transmitted founder virus (TFV);

^e^ Date post infection (number of days between estimated date of infection and sample collection for immunogenicity testing);

^f^ Log Plasma HIV-1 RNA (copies/ml);

^g^ Absolute CD4 T cell counts (cells/ml).

### A majority of CD8 T cells during chronic HIV-1 infection can cross-recognize both non-adapted and adapted epitopes

**Fig 3:**
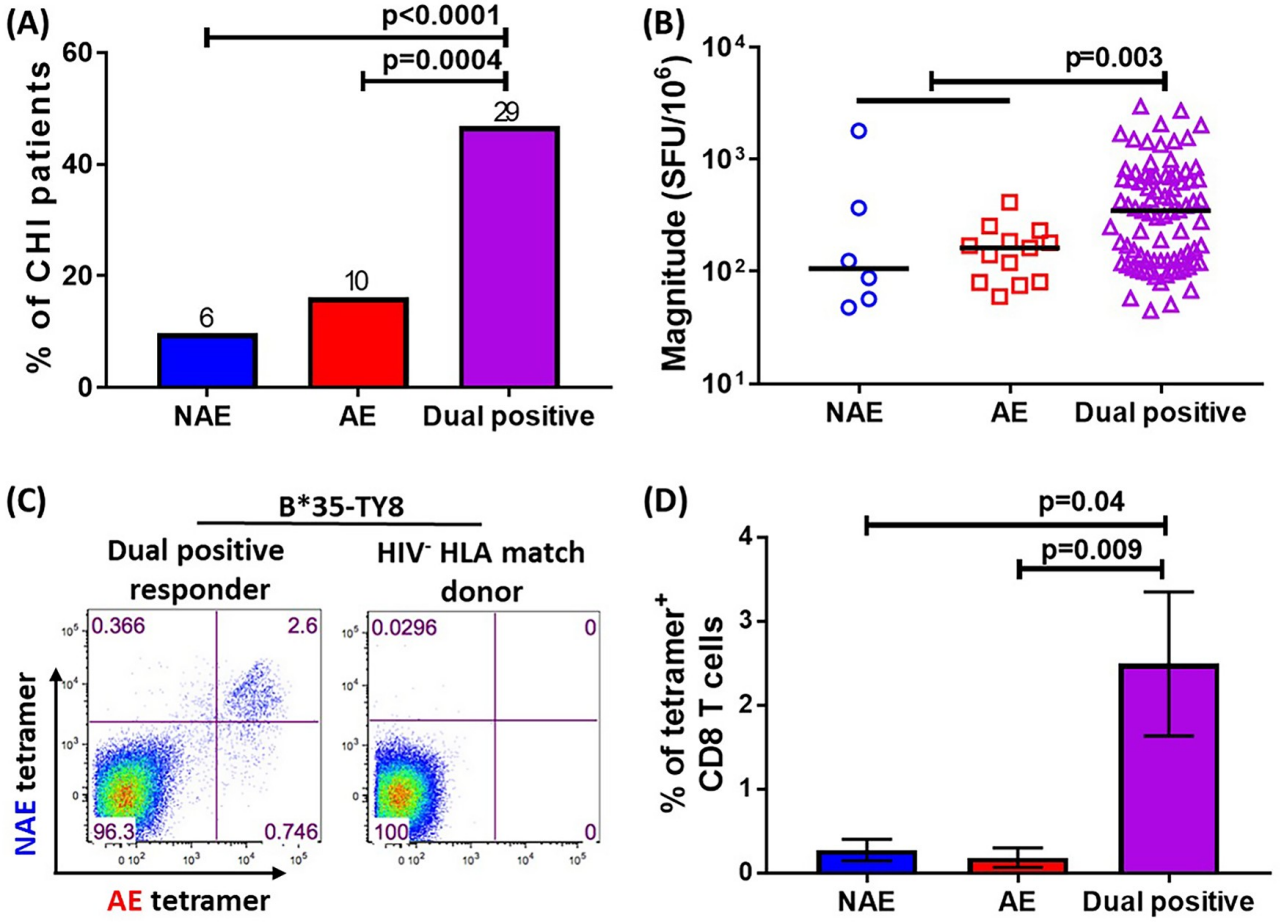
Cross-reactive CD8 populations that recognize both NAE and AE are common during chronic HIV infection (CHI). **(A)** Percentage of patients tested in cross-sectional study (N = 62) responding to both NAE and AE (dual positive) or either (single positive) at 10uM. The number of patients showing dual or single positive responder is shown on top of each bar. **(B)** Magnitude of dual positive responses and single positive responses (N = 118) were compared. Each dot represents a single NAE and/or AE response. **(C)** Representative flow cytometry plots of PBMC sample obtained from a B*3501-TY8 NAE/AE dual positive CHI patient stained by B*3501 tetramer pairs is shown. PBMC from HIV negative B*3501 expressing donor was used as negative control. **(D)** Cumulative data from six CHI patients is shown. Four different tetramer pairs were used in this assay as described in Methods. The frequency of tetramer positive CD8 T cell population for all patients was normalized to HIV negative donors. To determine the statistical significance, Fisher’s exact test was used in **(A)** and Mann–Whitney U test was used in **(B and D)**.

Prior work by our group showed a broadening of CD8 T cell cross-reactivity from acute to chronic infection [16] and our IFNγ ELISpot data showed an increase in AE responses in chronic infection. We, therefore, determined whether the same population of CD8 T cells in chronic infection would respond to both the NAE and AE forms of an epitope. A higher proportion of patient samples responded to both the NAE and AE (dual positive response) as compared to only the NAE or AE form (single positive response, p<0.0001 and p = 0.0004 respectively, Fig 3A). These dual positive responses were also greater in magnitude as compared to single positive responses (combination of single NAE and AE responses, p = 0.003, Fig 3B). Next, we stained these cells with four pairs of NAE and AE specific HLA-I tetramers conjugated with different fluorochromes (NAE-APC or AE-PE). In all six dual responding individuals tested, we consistently observed a single population of CD8 T cells labeled by both the NAE and AE tetramers as shown in a representative example in Fig 3C and cumulatively in Fig 3D, indicating a dominance of cross-reactive CD8 T cells responding to both NAE and AE during chronic infection.

### CD8 T cells expanded *in vitro* by adapted epitopes exhibit higher cytotoxicity against CD4 T cells during chronic HIV-1 infection

**Fig 4:**
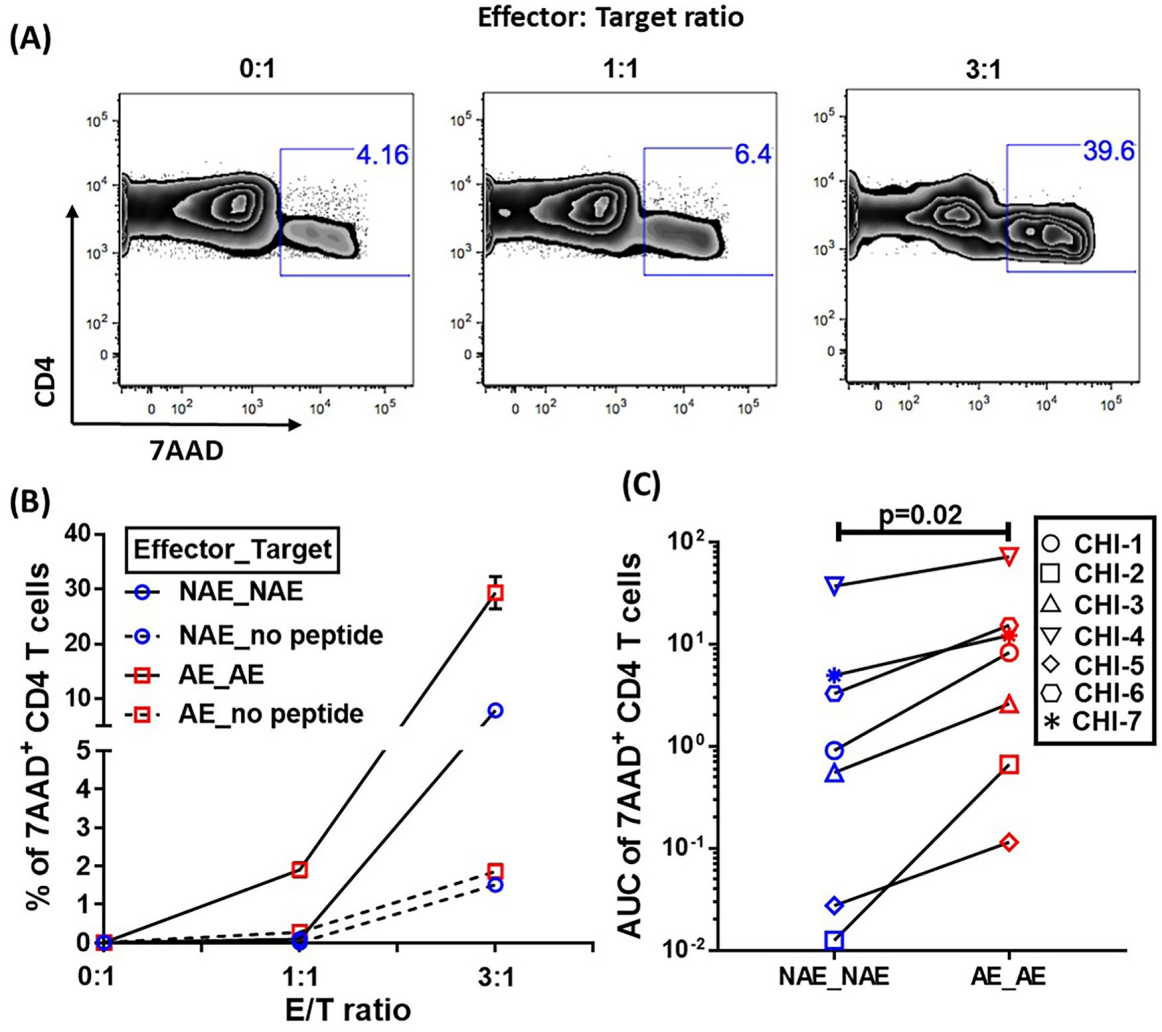
AE-specific CD8 T cells are more cytotoxic to CD4 T cells than NAE-specific ones as measured by level of target cell apoptosis. **(A)** Representative flow cytometry plots showing the accumulation of 7 AAD in peptide pulsed CD4 T cells after incubation with AE-specific CD8 T cells at E:T ratios of 0:1, 1:1 and 3:1. **(B)** For each E:T ratio, the average percent of 7AAD positive CD4 target cells (duplicates) was plotted for each co-culture condition. The data from E:T ratio (0:1) i.e. no CD8 T cell was normalized to 0% 7AAD positive cells and subtracted from the percentage of 7AAD+ cells with E:T ratios of 1:1 and 3:1. **(C)** Cumulative data from 7 CHI patients is shown. The area under the curve (AUC) was calculated using Prism7 and used to quantify the CD4 target killing capacity of peptide specific CD8 lines. Each dot represents killing capacity of each peptide specific CD8 line while each line represents a paired NAE and AE comparison for each individual. Wilcoxon matched-pairs signed rank test were used to determine statistical significance in **(C)**.

Since we observed increased AE-specific IFNγ responses in chronic infection and these responses could often be attributed to cross-reactive CD8 T cells, we evaluated the cytotoxicity of CD8 T cells recognizing AE versus NAE pulsed targets. We expanded antigen-specific cells *in vitro* by co-culturing the isolated CD8 T cells with peptide pulsed autologous monocytes. Using these peptide-specific CD8 T cell lines generated against NAE or AE, we assessed cytotoxicity with a 7AAD killing assay, in which we quantified the percentage of 7AAD^+^ CD4 T cell targets at various effector to target ratios as an output of CD8 T cell cytotoxicity, as described previously [14, 16]. Overall, cytotoxicity was assessed for six different NAE/AE pairs in seven CHI patients (S3 Table). A representative example of flow cytometry based gating and normalized data from CHI-6 is shown in S1A Fig, Fig 4A and 4B. Cumulative data analysis showed that the CD8 T cell lines generated against AE consistently elicited stronger cytotoxic responses to peptide-pulsed CD4 T cells (p = 0.02) than their corresponding NAE counterparts (Fig 4C) even though their *ex vivo* IFNγ ELISpot response magnitude were not significantly different (S2A Fig). Whenever cell number was not limited, we also tested these CD8 T cell lines for cytokine/effector molecules production, including IFNγ, TNFα, CD107a, perforin, and granzyme A/B production, which have been shown to be relevant to CD8 T cell cytotoxicity [26–28]. We did not detect any significant differences in the frequency of their production (either mono or polyfunctional responses) between NAE and AE specific CD8 T cell lines (S2B–S2F Fig).

### CD8 T cells stimulated with adapted epitopes express a less exhausted and senescent phenotype

**Fig 5:**
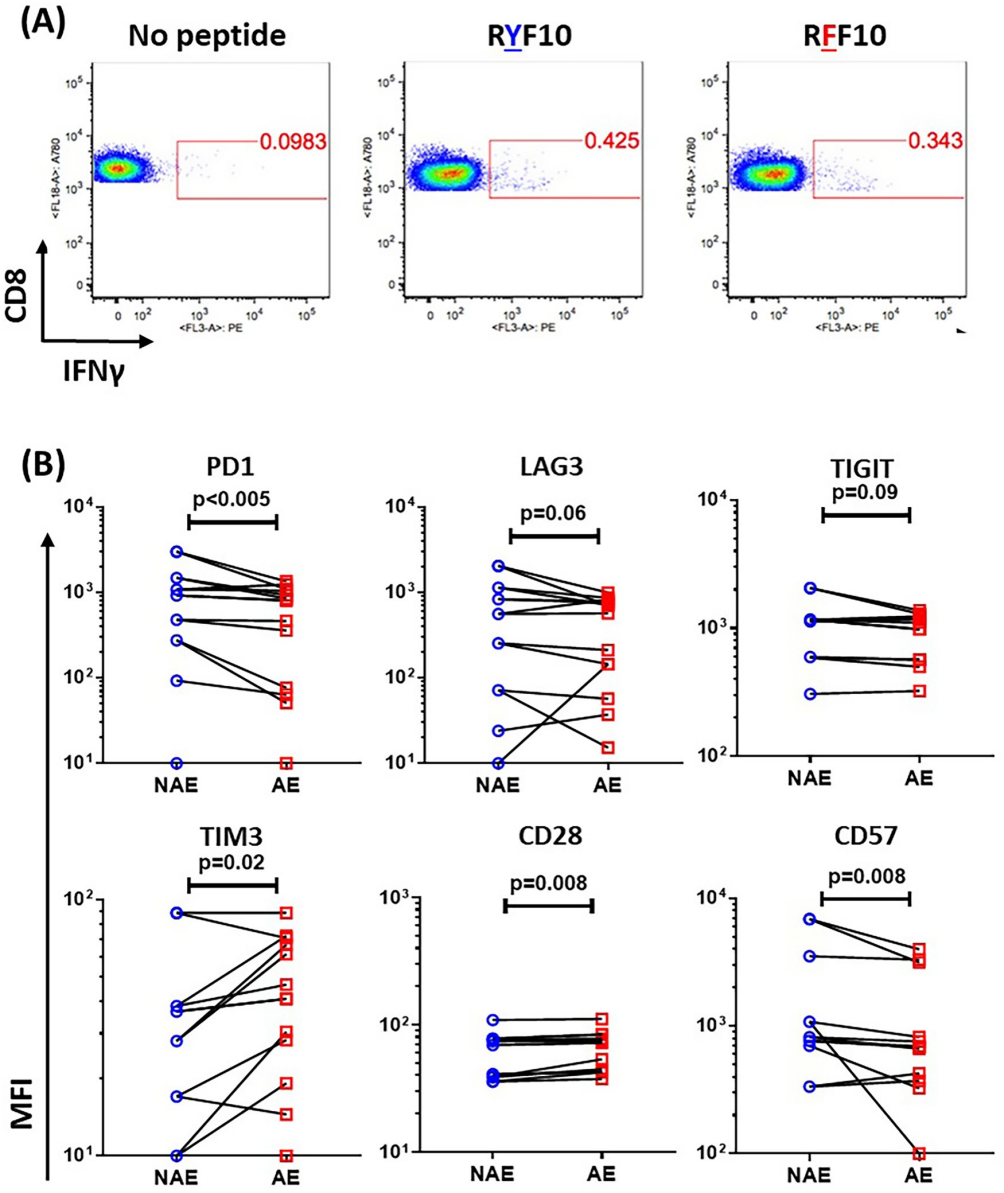
Differential expression of markers of immune activation and exhaustion. Dual positive PBMCs were stimulated with either NAE or AE for 12 hrs. PBMC were stained by anti-IFNγ antibody and measured for surface marker expression on antigen specific CD8s. **(A)** Representative flow cytometry plots showing IFNγ positive CD8s responding to NAE- RYF10 (RYPLTFGWCF) or AE-RFF10 (RFPLTFGWCF). **(B)** Cumulative data for 14 epitope pairs showing median fluorescence intensity (MFI) of PD1, LAG3, TIGIT (wasn’t tested for 2 epitope pairs due to limited cell availability of PBMC from 1 patient), TIM3, CD28 and CD57 markers on IFNγ positive CD8 T cells. Wilcoxon matched-pairs signed rank test were used to determine statistical significance.

Since we saw differences with cytotoxicity but not with cytokine/effector function, we next asked if these CD8 T cells respond differently to stimulation by NAE versus AE. Multiple surface markers, including PD1, TIM3, LAG3, TIGIT, CD160, CD27, CD28, CD38, CD57 and CD69, have been shown to play an important role in regulating CD8 T cell function and impacting disease progression during HIV-1 infection [29–37]. Thus, we assessed the expression level of these surface makers. Because we had previously observed that a single CD8 T cell population was responsible for dual NAE and AE responses, we assessed the expression of these markers on IFNγ^+^ CD8 T cells following NAE or AE peptide stimulation (S1B Fig, Fig 5A). CD8 T cells stimulated with AE expressed significantly lower levels of PD1 and CD57 and higher levels of CD28 (Fig 5B), suggesting a more activated and less exhausted/senescent phenotype consistent with the enhanced cytotoxicity data seen in Fig 4C. We also observed a trend towards lower expression of the other two exhaustion markers, LAG3 and TIGIT, on CD8 T cells responding to AE (Fig 5B). While increased expression of TIM3 was seen on AE-responding CD8 T cells (Fig 5B), a recent study found no evidence that TIM3 truly marks exhausted CD8 T cells [38]. Overall, these data indicate that cross-reactive CD8 T cell responses against NAE and AE are associated with a differential expression of molecules involved in CD8 T cell function and that AE-stimulated CD8 T cells have a less exhausted and less senescent phenotype.

### Viral quasispecies in chronically infected individuals encode a high frequency of adapted epitopes

Since AE were increasingly targeted and induced a higher cytotoxic response during chronic infection, we next determined whether the virus evolved by mutating away from this increased immune pressure during chronic infection. We sequenced the viral quasispecies present in chronic infection in six of the seven individuals that were also evaluated for cytotoxicity (Fig 4). The seventh individual (CHI-3) had undetectable viral load at the time point of interest preventing successful sequencing attempts.

**Fig 6:**
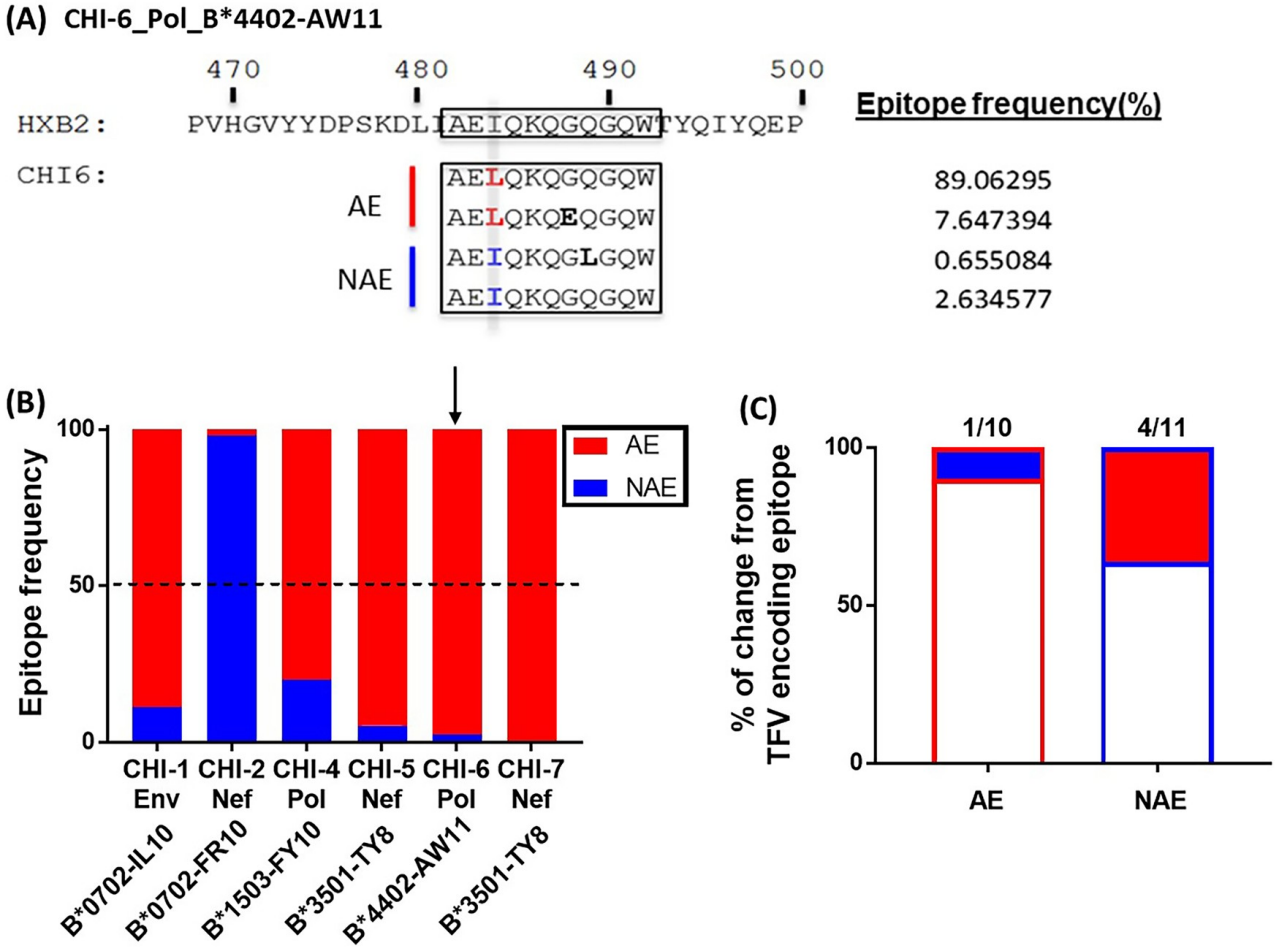
Adapted epitopes are encoded in a significant proportion of viral quasispecies during chronic HIV infection (CHI). **(A, B)** Viral sequencing was attempted from the plasma samples of all 7 CHI patients in Fig 4 and successful in 6 of the 7. The sample from CHI-3 was not successful due to undetectable viral load. **(A)** Representative example showing alignment between viral sequences, HXB2 sequence and the epitope of interest. Both the sequence of each version of the epitope as well as the epitope frequency was identified, an example of which is shown here. **(B)** The epitope frequency of NAE and AE was calculated for each patient viral quasispecies. **(C)** Viral sequences were obtained from 5 AHI patients at both acute (Mean DPI = 37) and chronic infection (Mean DPI = 511). HIV evolution on an epitope level was determined during acute and again during chronic infection. The frequency of AE evolving to NAE (1/10) or from NAE to AE (4/11) during this period of time is shown. Fisher’s exact test was used in (**C**) to determine statistical significance.

Phylogenetic analyses showed significant viral heterogeneity at the quasispecies level among all six individuals. For each individual, the frequency of NAE and AE of interest was assessed with representative data shown in Fig 6A. A majority of HIV quasispecies encoded AE in five of the six individuals sequenced (Fig 6B). The exception is individual CHI-2, whose sequence revealed a higher frequency of the NAE-FRL9 (FPV**R**PQVPL) epitope (99.11%) as compared to its counterpart AE-FKL9 (FPV**K**PQVPL) epitope (0.89%) (Fig 6B).

We next determined whether viruses encoding AE are maintained over time. The chronic time point sequences were compared with the TFV sequences in the longitudinal cohort that we tested for TFV encoded epitope specific CD8 IFNγ response. Although AE responses were enriched over time as shown in Fig 2B and 2C, in the five patients in whom we sequenced and examined ten different AE, we only saw one case of mutation from AE to NAE (AE-FKL9 to NAE-FRL9). In the same group of patients, we saw four different cases out of eleven where NAE mutated to AE (36.36%, Fig 6C). Taken together, these data suggest that even though AE are increasingly recognized by CD8 T cells in chronic infection, they persist in circulating viral sequences and that AE may confer some yet to be described advantage to HIV-1.

### Adapted epitope-stimulated CD8 T cells facilitate dendritic cell maturation and HIV *trans*-infection

Our findings were puzzling since despite AE-specific CD8 T cells demonstrating a less exhausted phenotype and enhanced cytotoxicity, AE were the predominant epitope type in chronic infection. Indeed, due to their evolution in CHI, viral adaptations are defined by using chronic HIV sequences [11, 39]. A prior study by *Mailliard et al*. described impaired killing of dendritic cells by a variant epitope induced cross-reactive CD8 T cells [24]. DCs that came in contact with these cross-reactive CD8 T cells matured into a pro-inflammatory phenotype with an efficient viral *trans*-infection capacity. We thus hypothesized that during HIV-1 chronic infection, cross-reactive CD8 T cells responding to AE might promote DC maturation and facilitate HIV-1 *trans*-infection from DCs to CD4 T cells.

**Fig 7:**
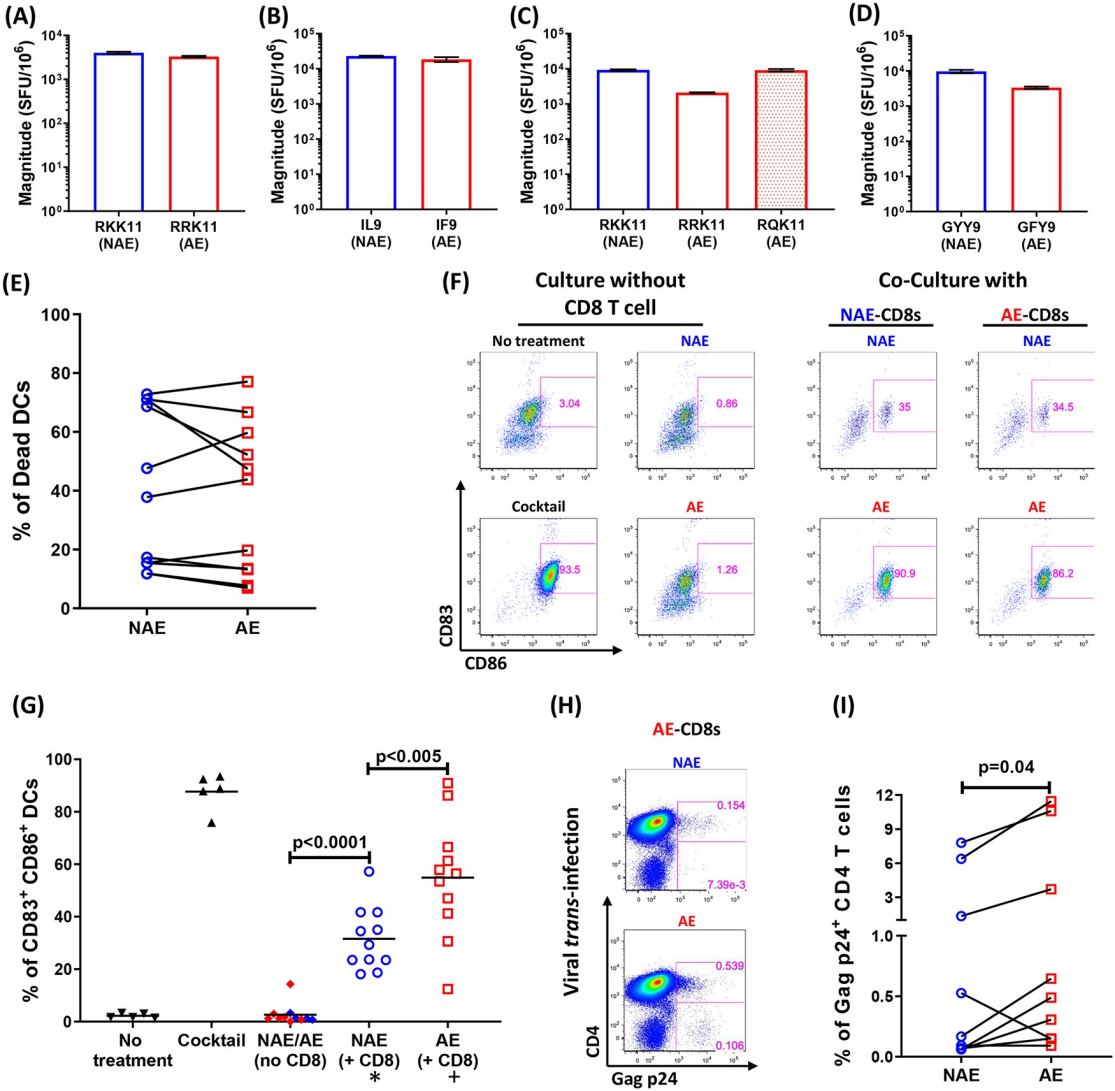
Increased DC maturation by AE-specific CD8 T cells may fuel HIV *trans*-infection. **(A-D)** Magnitude of IFNγ response to each indicated NAE or AE peptide from CD8 T cell lines generated from four CHI individuals is shown. **(E)** Immature DCs (iDCs) were generated from an HLA matched HIV naïve donor and then cultured with or without CD8 T cell lines pulsed with either NAE or AE. iDC cultured in maturation cocktail (as described in Methods) was used as positive control while iDC culture without treatment was used as negative control. Percentage of DC stained by dead cell dye (as described in method) is shown. **(F)** The expressions of CD83 and CD86 were measured on DCs cultured in each condition. **(G)** Frequency of mature DCs (defined by co-expression of CD83 and CD86) for each culture condition is shown. A total of 11 NAE/AE pairs is used for pulsing CD8 T cell lines (* represents CD8 T lines pulsed by NAE, while + represents CD8 T lines pulsed by AE, regardless of the epitope used for expansion). **(H)** iDCs were cultured with CD8 T cell lines pulsed with either NAE or AE. CD8 T cells were removed prior to loading DCs with HIV-1 virus (MOI = 10^−4^) and co-culturing them with activated autologous CD4 T cells. HIV Gag p24 expression was then measured in these CD4 T cells. **(I)** Cumulative data of viral *trans*-infection obtained from stimulation by 9 NAE/AE pairs is shown. Each dot represents the frequency of virally infected CD4 T cells resulting from a peptide stimulation. To determine statistical significance, Wilcoxon matched-pairs signed rank test was used in **(E)**, **(G**, comparing “CD8+NAE” and “CD8+AE” conditions) and in **(I)**. Mann–Whitney U test was used in (**G**, comparing “NAE/AE” and “CD8+NAE” conditions).

To test this hypothesis, we modified previously described DC maturation and *trans*-infection assays [24]. For validation experiments we used a cross-reactive SL9 (SL**Y**NTV**A**TL) CD8 T cell clone, derived from a healthy donor, that was able to cross recognize and respond to several natural variants including SFL9 (SL**F**NTV**A**TL) and SVL9 (SL**Y**NTV**V**TL) (S3A Fig) [40–42]. We observed a higher frequency of mature DCs (CD83^+^ CD86^+^) in the context of cross-reactive CD8 T cell responses to SFL9 and SVL9 as compared to the cognate response to SL9 (S1C and S3B Figs). We then cultured activated CD4 T cell with an R5-tropic virus at multiplicity of infection (MOI) of 10^−1^ and 10^−4^. Consistent with prior findings [43], an MOI of 10^−4^ was not sufficient to directly infect CD4 T cells (S3C Fig). Thus, matured DCs were incubated with virus at MOI of 10^−4^ for all subsequent viral *trans*-infection assays. When CD4 T cells were cultured with mature DCs co-cultivated with SFL9 and SVL9 pulsed CD8 T cells, we observed a higher frequency of Gag-p24 stained *trans*-infected CD4 T cells (S1D and S3D Figs), including T cells that had downregulated CD4 following infection, as has been previously described [44, 45]. Moreover, consistent with prior work [24, 43], we also observed more efficient viral infection, as measured by Gag-p24 expression, by DC-to-CD4 T cell *trans*-infection than from infection of CD4 T cells directly by free virus present in a supernatant (*cis*-infection) (p = 0.04, S3E Fig). We then used this optimized assay to test DC maturation in the presence of NAE and AE-generated CD8 T cell lines from PBMCs obtained from CHI patients with positive responses to both epitope forms. Additional responses to three NAE/AE groups in four patients, i.e. Gag A*0301-RLRPGGKK**K**YK (RKK11) / RLRPGGKK**R**YK (RRK11) / RLRPGGKK**Q**YK (RQK11), Env B*07-IPRRIRQG**L** (IL9) / IPRRIRQG**F** (IF9) and Nef A*3002- G**Y**FPDWQNY (GYY9) / G**F**FPDWQNY (GFY9), were tested. We first confirmed the functionality of CD8 T cell lines following epitope-specific expansion by testing for IFNγ production (Fig 7A–7D). Next, DC maturation assays were performed for each cell line to compare the DC phenotype in co-culture with NAE or AE stimulated CD8 T cells. When co-cultured with NAE or AE stimulated CD8 T cells, peptide pulsed DCs show no difference in cell death (Fig 7E). We also found that all CD8 T cell lines, regardless of the epitope used for expansion, resulted in a high frequency of mature DCs when stimulated with AE (p<0.005, Fig 7F and 7G), and these DCs demonstrated an enhanced ability to *trans*-infect virus to CD4 T cells (p = 0.04, Fig 7H and 7I). Taken altogether, our findings suggest that while there is a broadening of AE responses during chronic infection, these adaptations may contribute to viral pathogenesis by altering CD8 T cell function to facilitate DC-mediated viral *trans*-infection.

### CD8 T cells respond to adapted epitopes with lower antigen sensitivity

A lower antigen sensitivity, which is also often referred to functional avidity, was previously associated with the shift from cytotoxic to “helper-like” CD8 T cell phenotype which facilitated viral dissemination [24]. Thus, we hypothesized that CD8 T cells might respond to AE with a lower antigen sensitivity than NAE. PBMCs with paired NAE/AE responses on IFN-γ ELISpot were cultured with the relevant peptides at 10-fold serially diluted concentrations.

**Fig 8:**
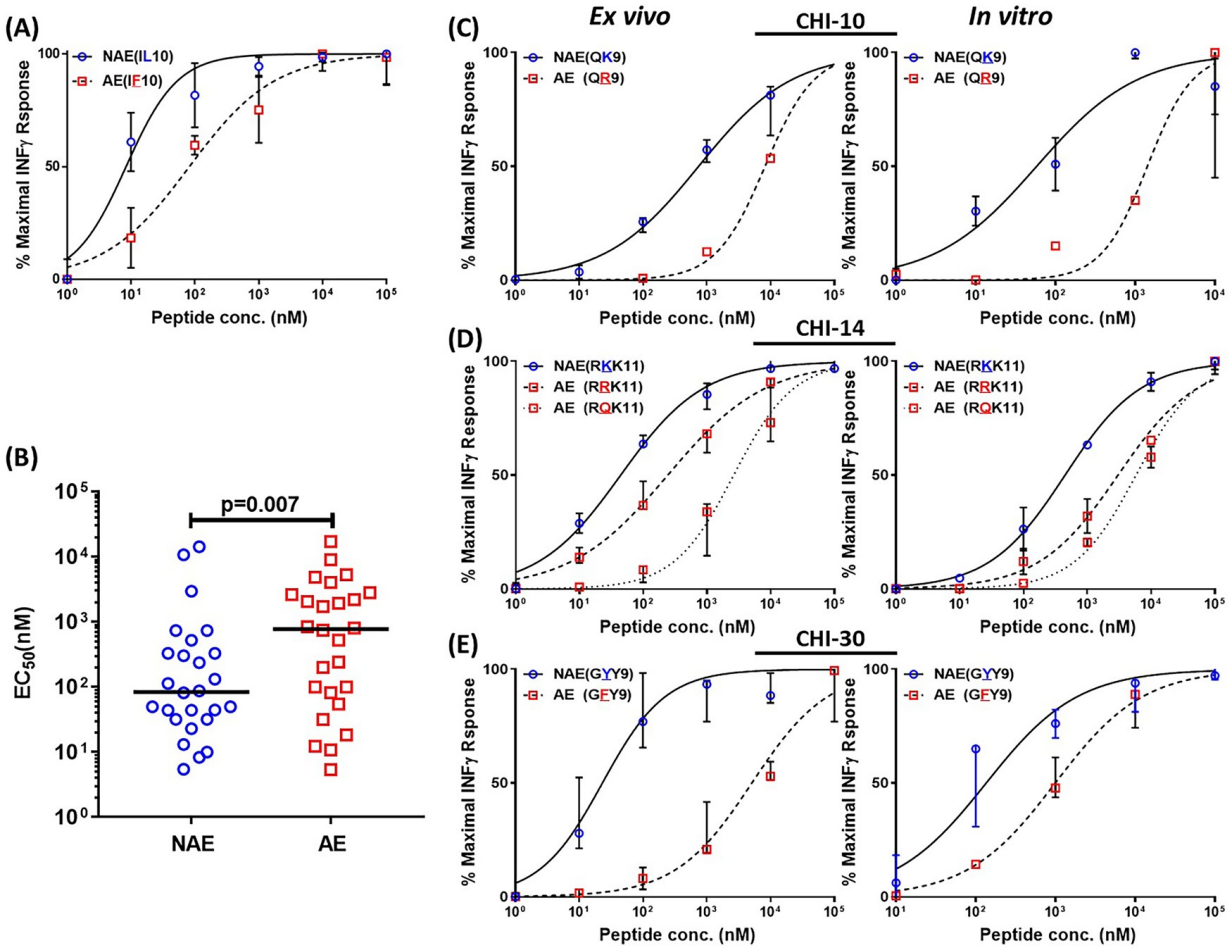
Lower *ex vivo* and *in vitro* antigen sensitivity of CD8-IFNγ responses against AE than NAE. **(A)** Five to six log fold serial dilutions of peptides were used in an IFN-γ ELISpot assay depending on the cell availability. A representative example of a six log fold serial dilution assay is shown. Each curve represents an epitope specific response. The EC50 value (peptide conc. eliciting 50% of maximal response) for each curve was calculated using Prism7. **(B)** Cumulative data for 26 epitope pairs in 16 CHI individuals screened as in **A** is shown. (**C-E**) Antigen sensitivity data of PBMCs (*ex vivo*) and peptide specific CD8 T cell lines (*in vitro*) from dual positive responders is shown. The mixed effects model was used to determine statistical significance in (**B**).

In total, 26 NAE/AE pairs from sixteen CHI individuals were tested *ex vivo*. The CD8 T cell responses to AE showed a 9-fold higher EC50 (median = 773.6) than NAE (median = 84.42). These data showed that AE-specific responses had a lower antigen sensitivity or needed a higher antigen concentration than NAE ones (p = 0.007, Fig 8A and 8B), in spite of comparable *ex vivo* IFNγ ELISpot responses (S4 Fig). Because our DC maturation and CD4 *trans*-infection assays were performed using CD8 T cells *in vitro*, we also performed antigen sensitivity testing using expanded CD8 T cells and demonstrated that AE-specific CD8 T cells consistently needed higher antigen concentration for stimulation (lower antigen sensitivity) than their NAE counterparts (Fig 8C–8E).

## Discussion

Earlier studies have mainly focused on HIV adaptation in the context of a lack of immune recognition through abrogated HLA-I binding and/or impaired CD8 T cell receptor (TCR) recognition [46–49] or classical escape. These studies were also often limited to certain immunodominant epitopes restricted by HLA-I alleles that are linked to delayed disease progression [9, 50–52]. It is worth noting that infection with viruses encoding classical escape mutations to these protective alleles results in a loss of viral control [13, 50, 53]. Indeed, we previously showed that the effect of protective HLA-I alleles, such as HLA-B*57, was abrogated by infection with a virus pre-adapted to these alleles [14, 54]. We also found that AE were poorly immunogenic in acute HIV-1 infection, indicating classical escape and providing a possible explanation as to why infection with a virus pre-adapted to a host’s HLA-I alleles predicts disease progression. However, a growing body of literature suggests there are non-classical forms of HIV adaptation where immune recognition is preserved but some other advantage is conferred to the virus [2, 23]. Our current study illustrates one such mechanism where, in spite of persistent immune recognition induced by HLA-I associated adapted epitopes, the resulting CD8 T cell response appears to aid viral *trans*-infection of CD4 T cells by promoting dendritic cell maturation.

Although a population based study on HIV-1 subtype C estimated that roughly half of HLA-associated adaptations impact peptide binding or HLA processing [55], we do not observe a significant difference in predicted HLA-I binding affinity between our predicted NAE and AE [14, 56]. Additionally, the majority of HLA-I associated adaptations in our studies were not located in HLA-I anchor sites (S1 Table). This would suggest that most predicted HLA-I adaptations could potentially be recognized by CD8 T cells, a prediction that we confirmed in our studies. Our current study shows that AE-specific responses increased in magnitude and frequency from acute to chronic infection, suggesting that CD8 T cell responses to AE take longer to develop, perhaps due to a lower sensitivity of CD8 T cells to AE. Besides, in contrast to our prior finding where AE specific CD8 T cells harbor a poor cytotoxicity during acute infection, we demonstrated a greater cytotoxicity activity of AE specific CD8 T cells in chronic infection. This difference might be explained by differing levels of exhaustion/senescence and/or other contributing factors, like viral load, epitope frequency and CD8 T cell clonal profile. However, our understanding of what factors influence the kinetics of the CD8 T cell response, as well as how such kinetics are connected to HIV-1 adaptation, remains incomplete. Past studies focusing on the expansion of CD8 T cell responses in HIV infection illustrated the dynamics of antigen-specific TCR repertoires [57, 58], and future work should delve into how changes in TCR repertoires may influence the development of responses to AE versus NAE as well as the evolution of viral adaptation.

Another intriguing finding in this study was that many of the NAE and AE responses detected were due to cross-reactive CD8 T cells. While some mono-specific CD8 T cell responses were detected, they were consistently of a lower magnitude as compared to the cross-reactive ones. Although this is in contrast to a previous study indicating that CD8 T cell responses to escape variant resulted from *de novo* CD8 T cell populations [15], our findings agree with our prior study showing that HIV-specific CD8 T cell cross-reactivity is enhanced during chronic infection [16]. Moreover, several other studies have demonstrated the presence of cross-reactive CD8 T cell responses in chronic HIV infection [16, 24, 59–63]. In addition, in SIV infection, cross-reactive CD8 T cell responses to variant epitopes arise over time but fail to control escaped viral quasispecies [18]. Indeed, our sequencing analysis indicated that a majority of viral quasispecies in chronic infection encode AE despite our observation of enhanced cross-reactive responses and higher *in vitro* cytotoxicity of AE-specific CD8 T cells, suggesting that these CD8 T cell responses may be unable to effectively control virus *in vivo*. Furthermore, a recent study found that AE within TFV Gag sequences are unlikely to revert to NAE or mutate to another variant [64].

CD8 T cells can play a “helper” role that impacts the overall immune response and anti-viral immunity [65–67]. For example, besides killing virally infected cells, CD8 T cells can also induce lysis of antigen presenting immature dendritic cells (iDCs) [65] and promote DCs maturation in viral infection [67, 68]. In the context of HIV, we showed that cross-reactive CD8 T cells from chronically infected individuals, who responded to AE more efficiently, induced greater DC maturation than the same CD8 T cell population responding to NAE. These mature dendritic cells more efficiently *trans*-disseminated HIV to activated CD4 T cells. Thus a positive feedback loop is established between CD8 T cells and DC as more AE specific “helper” CD8 T cells are primed by preferentially matured AE-expressing DC. As a consequence, an “epitope spreading” phenomenon aiding in pathogenesis could be exploited by HIV-1. The persistence of AE-encoded viral quasispecies and increasing AE specific CD8 recognition in chronic infection could be explained, at least partially, by this “helper” role of CD8 T cells.

The persistence of AE-encoded viral quasispecies suggest the “helper” rather than the “killer” effect as a better predictor of CD8 T cell potency *in vivo*. However, it remains unclear why AE-specific CD8 T cells exhibited greater cytotoxicity toward target CD4 T cells while promoting DC maturation to fuel viral infection. A recent study showed resistance of monocyte-derived macrophages to CD8 T cell killing was associated with prolonged cell-to-cell contact that subsequently led to a pro-inflammatory environment suboptimal for effector cell function. Meanwhile, the same CD8 T cells rapidly killed CD4 T cell targets [25], suggesting that differential pathways associated with susceptibility or resistance to effector cell killing can occur when interacting with different target cells. Another past study showed an association between the optimal quantity (antigen concentration) and quality (peptide-MHC stability) of antigen and a faster transition into “phase two” CD8 T cell-DC interaction which is known to be necessary *in vitro* for full commitment to T cell activation [69]. It has also been shown that CD8 T cell derived Granulocyte-Macrophage Colony-Stimulating Factor (GM-CSF) plays a critical role in facilitating DC maturation and production of pro-inflammatory cytokines [68]. It is possible that an analogous phenomenon is occurring in the context of AE-specific CD8 T cell responses. Our findings indicate that AE induce CD8 T cell responses with a higher antigen threshold than those induced by NAE, which may result in a prolonged synapse time between effector CD8 T cells and targets such as DCs, ultimately leading to a detrimental pro-inflammatory environment that fuels infection [24, 25]. It is worth investigating in future studies how this process might tip the balance in favor of DC-mediated viral *trans*-infection over killing of CD4 T cells.

In summary, we show the presence of enriched AE-specific CD8 T cell responses in chronic HIV infection and demonstrated that these responses contributed to enhanced viral *trans*-infection rather than viral containment. This study expands our current understanding of how HIV exploits host immune responses in chronic infection and highlights the importance of understanding AE-specific CD8 responses in the context of vaccine and therapeutic strategies. For instance, future vaccine strategies, especially those aiming at inducing broader CD8 T cell responses by targeting multiple variants, should be designed with caution. Additionally, many HIV-1 infected individuals are not diagnosed until chronic infection, and recent studies have shown that the latent reservoir in chronically infected individuals likely encodes CD8 T cell escape mutations [70]. While our studies are hopeful in that the majority of AE are immunogenic in chronic infection, they also indicate that these CD8 T cells may be continuing to drive the infection of CD4 T cells. As such, future studies may need to focus on a better understanding and improvement of AE-specific CD8 T cells as part of a comprehensive strategy towards HIV cure.

## Materials and methods

### Ethics statement

All patients included in this study were adults and recruited from the University of Alabama at Birmingham Adult AIDS 1917 clinic after obtaining written, informed consent and approval from the IRB (X981027004, X160125005 and X140612002) at UAB.

### Patient cohorts

All patients were typed for their HLA class I alleles. Peripheral blood mononuclear cell (PBMC) and plasma samples were collected. Samples from acutely HIV-1 infected (AHI) patients naïve to antiretroviral therapy (ART) at an average of 37 days post-infection (DPI) (n = 13) were tested. Transmitted founder virus (TFV) sequences were inferred from the plasma of these 13 AHI patients using a single genome amplification method, as described previously [71]. Longitudinal chronic infection samples from these patients at an average of 511 DPI were also tested. An additional cross-sectional cohort of chronically HIV-1 infected (CHI) patients infected at least one year (n = 65), were studied.

### Epitope selection and peptide synthesis

We predicted the optimal sequences (8-11mer) of HLA-I restricted non-adapted (NAE) and adapted epitopes (AE) using Microsoft Research’s EPIPRED software (S1 Table) [14]. Autologous peptides were designed for each AHI patient based on HLA-I alleles and TFV sequence. For each CHI patient, both NAE and the corresponding AE peptides were determined based on HLA-I alleles. Overall, 77 NAE/AE groups (31 restricted by HLA-A, 41 restricted to HLA-B and the 5 pairs restricted by HLA-C alleles) were tested in this study. All peptides were synthesized in a 96 well array format (New England Peptide). Each peptide was reconstituted at 40 mM in 100% DMSO and stored at -70°C [14, 16].

### IFNγ ELISpot

Nitrocellulose plates (Millipore) were coated overnight with anti-IFNγ antibody and were subsequently blocked with R-10 media (RPMI + 10% human AB serum) for 2h. PBMCs were thawed and rested overnight at 37°C/5% CO2. PBMCs (10^5^ cells/well) were cultured in duplicate (when cell number was limited) or triplicate with the peptide of interest at 10 μM in R-10 media for 22-24h. Cells cultured in media without peptide and in media with PHA were used as negative and positive controls, respectively. Following incubation, the plates were washed and treated with biotinylated anti-IFNγ antibody for two hours followed by streptavidin-alkaline phosphatase for one hour, and finally developed with the NBT/BCIP substrate for 5–10 minutes. Plates were read and counts were determined by CTL ImmunoSpot analyzer (version 5). Number of spots was averaged and normalized to SFU (spot forming units) per 10^6^ cells (SFU/10^6^). A positive response was defined as ≥55 SFU/10^6^ and ≥ four times background (media only wells) [6, 14, 16].

### Antigen sensitivity

Serial 10-fold dilutions (from 10^6^ to 10^0^ nM) of peptides were used to stimulate PBMCs in an IFNγ ELISpot assay as described above (done in triplicate). Antigen sensitivity or functional avidity was then quantified as an EC50 value [61, 62, 72], which is the peptide concentration that elicited 50% of maximal IFNγ response for any given epitope. This value was calculated by plotting a dose-response curve in GraphPad Prism (version 7.0).

### HLA class I tetramers

Four paired NAE/AE based HLA class I tetramers were synthesized by NIH Tetramer Core Facility as follows: A*23:01-RYF10 (RYPLTFGWCF)/ RFF10 (RFPLTFGWCF), B*07:02-NRI10 (NPRISSEVHI)/ HKI10 (HPKISSEVHI), B*35:01-TIY8 (TPGPGIRY)/ TVY8 (TPGPGVRY) and B*44:02-AIW11 (AEIQKQGQGQW)/ ALW11 (AELQKQGQGQW). All NAE and AE tetramers were conjugated to APC and PE, respectively. Each tetramer was validated in an individual with a positive IFNγ ELISpot response to the epitope of interest and HIV^+^ HLA-I mismatched and HIV^-^ HLA-I matched PBMC were used as negative controls. Tetramer titrations were performed using two-fold dilutions to ascertain the optimal concentration, which was then used in all subsequent assays. Of note, certain HLA subtypes, e.g. B*0702, B*3501, and B*4402, were enriched since responses directed by these HLA-restrictions were used for tetramer analysis. A positive tetramer population is defined as > 3 fold than the negative control and ≥ 0.05% above the background. Besides, the reactive tetramer staining should be significantly higher than the negative control based on Fisher’s exact as adapted from prior study [73].

### *Ex vivo* tetramer staining

PBMCs with dual NAE and AE specific CD8 T cell responses were labeled with tetramers at room temperature for 30 min and then were stained at 4°C for 30 min with dead cell dye (Invitrogen), anti-CD3-Alexa 780 (eBioscience), anti-CD4-Qdot655 (Invitrogen), and anti-CD8-V500 (BD Pharmingen). At least 10^6^ total events were acquired on an LSR II flow cytometer (BD Immunocytometry Systems), and data were analyzed using FlowJo (version 9.6.4; TreeStar Inc.).

### Phenotyping for markers of immune activation/exhaustion

PBMCs responding to both NAE and AE in an IFNγ ELISPOT assay were pulsed with the peptides at 10μM in the presence of anti-CD28 and anti-CD49d. Monensin and brefeldin A were added 1 hour after peptide stimulation. The cells were incubated for an additional 11h. Following incubation, cells were surface stained for 30min at 4°C with dead cell dye (Invitrogen), anti-CD3-Alexa 780 (eBioscience), anti-CD4-Qdot655 (Invitrogen), and anti-CD8-V500 (BD Pharmingen) in the following panels: (1) anti-TIGIT-Percp/CY5.5 (Biolegend), anti-CD160-Alexa488 (eBioscience), anti-PD1-Alexa700 (Biolegend), anti-TIM3-BV421 (Biolegend) and anti-LAG3-PECy7 (Biolegend) and (2) anti-CD28-FITC (BD Pharmingen), anti-CD27-PECy7, anti-CD38-v450 (eBioscience), anti-CD57- Percp/CY5.5 (Biolegend), and anti-CD69-Alexa700 (Biolegend). The cells were then permeabilized and stained with anti-IFNγ-PE at 4°C for 30 min. At least 10^6^ total events were acquired on an LSR II flow cytometer (BD Immunocytometry Systems), and analyzed using FlowJo (version 9.6.4; TreeStar Inc.). The criteria of positivity is the same as defined above for tetramer staining [73].

### *In vitro* expansion of CD8 T cells

Epitope-specific CD8 T-cell lines were expanded *in vitro* as previously described [14]. Briefly, cryopreserved PBMCs (obtained from chronically HIV-1 infected patients) were thawed and plated in a 48-well plate at 1.2×10^6^ cells/ml in serum free RPMI media. Plates were incubated at 37C/5% CO_2_ for two hours, after which media containing non-adherent cells was removed. Adherent cells were irradiated at 3,000 rad and pulsed with the appropriate peptide at 10 μM for 2 h. Autologous CD8 T cells were isolated from the same PBMC sample using the CD8 untouched isolation kit (MACS Miltenyi Biotec). CD8 T cells were then plated at 0.5×10^6^ cells/well onto the peptide-pulsed monocytes in the presence of complete media (RPMI+10% FBS) containing IL-7 (25 ng/ml). IL-2 (50U/ml) was added to the culture on the second day. The culture was then maintained by replacing half the media with freshly made media containing IL-2 (50U/ml) every three days, and CD8 T cells were re-stimulated on day seven (and weekly thereafter) with peptide-pulsed monocytes. CD8 T cell clone (SL9) was a gift from Dr. June Kan-Mitchell.

### Intracellular cytokine staining (ICS)

Cytokine and effector molecule production was measured using flow cytometry as described previously [16]. Briefly, 0.5×10^6^ epitope-specific CD8 T cell lines were stimulated with cognate peptide at 10 μM in the presence of anti-CD28 and anti-CD49d as well as anti-CD107a-FITC (BD Biosciences) antibodies. Monensin and Brefeldin-A (BD Biosciences) were added one hour after peptide stimulation, and the cells were incubated for an additional 5 hours (CD8 T cell lines) at 37°C/5% CO_2_. Following incubation, cells were stained with dead cell dye (Invitrogen), anti-CD3-Alexa 780 (eBioscience), anti-CD4-Qdot655 (Invitrogen), anti-CD8-V500 (BD Pharmingen), anti-CD14-Percp/CY5.5 (BD Pharmingen) and anti-CD19-Percp/CY5.5 (BD Pharmingen) at 4 °C for 30 min. Cells were then permeabilized and intracellularly stained with anti-IFNγ-Alexa 700 (BD Biosciences), anti-TNFα-PECy7 (BD Biosciences), anti-Perforin-PE (eBioscience) and anti-Granzyme A/B-V450 (BD Biosciences) at 4°C for 30 min. At least 300,000 total events were acquired on an LSR II flow cytometer (BD Immunocytometry Systems), and data were analyzed using FlowJo (version 9.6.4, TreeStar Inc.). The criteria of positivity is the same as defined above for tetramer staining [73]. Polyfunctionality analysis was performed using boolean gating and polyfunctionality index was calculated using the method as described previously [74]. Briefly, we used algorithm (1)

$$\text{Polyfunctionality}\;\text{index}=\sum_{i=0}^{5}Fi*{\left(\frac{i}{5}\right)}^{q}$$ (1)

Where 5 is the number of functions studied as described in this assay, *Fi* is the frequency of cells displaying *i* functions and *q* is the parameter that modulates the weight of each *Fi*. In this study, we used *q* = 1 (the most conservative value).

### *In vitro* cytotoxicity assay

Epitope-specific CD8 T cells were expanded as described above and rested in R-10 media without IL-2 for 24 hours at 37°C/5%CO_2_. Target CD4 T cells were isolated from HIV-1 seronegative individual matched for the HLA-I allele of interest and were activated with PHA (5μg/ml) and IL2 (50U/ml) for two days. Activated target cells were cultured with or without cognate NAE or AE peptide for one hour. Next, NAE or AE specific CD8 T cell lines were co-cultured with CD4 T cell targets in duplicate at 0:1, 1:1, and 3:1 effector to target (E/T) ratios for 24 hours. After incubation, the co-cultured cells were surface stained with anti-CD3-Pacific Blue and anti-CD4-Qdot655 (all Invitrogen) at 4°C for 30 min. The cells were then labeled with 7-aminoactinomycin D (7AAD, BD Biosciences) at 5pg/μl for 30 min at 4°C. Events were acquired on an LSR II flow cytometer to detect the apoptosis of CD4 T cells (7AAD^+^ CD4 T cells) as an indication of CD8 T cell mediated killing. Data for each E:T ratio was normalized to corresponding negative control at the same E:T ratio and then normalized to E:T at 0:1 for each line. The area under curve (AUC) value was calculated for the 7-AAD expression using GraphPad Prism (version 7.0) and was used for statistical analysis.

### Generation of monocyte derived dendritic cells (moDC)

Monocytes were isolated from PBMCs (obtained from chronically HIV-1 infected patients) using the human CD14 MicroBeads (MACS Miltenyi Biotec) and cultured for 7 days in IMDM (Invitrogen) media containing 10% FBS in the presence of GM-CSF and IL-4 (both at 1000 IU/ml; R&D Systems) to generate immature DC (iDC). Half of the media was replaced every two days with freshly made media containing 1000 IU/ml of GM-CSF and IL-4 to maintain the DC culture.

### DC maturation assay

Epitope-specific CD8 T cells were added directly to autologous iDC at 3:1 effector to target (E/T) ratio in the presence or absence of peptide of interest (10 μM) and co-cultured for 48 hours. Immature dendritic cells cultured in the presence of a maturation cocktail containing TNFα (50ng/ml), IFNα (3000U/ml), IFNγ (1000U/ml), IL-1B (25ng/ml), and pI:C (20ug/ml) were used as a positive control. After two days, cells were stained with dead cell dye (Invitrogen), anti-CD3-Pacific Blue, anti-CD8-V500, anti-CD14-alexa700, anti-CD83-PE, and anti-CD86-FITC (all from BD Pharmingen) at 4°C for 30 min. Cells were then washed and events were acquired on an LSR II flow cytometer.

### HIV-1 *trans*-infection assay

An HIV-1 infectious molecular clone (IMC) was generated using the sequence of a transmitted founder virus (HIV-TRJO) as a viral backbone (provided by Dr. Christina Ochsenbauer). DC were co-cultured with CD8 T cells in the presence of peptide of interest or maturation cocktail (see above) for 48 hours. CD8 T cells were then removed from the culture using CD8 Dynabeads (Invitrogen), and the DC were loaded with a low MOI of virus (10^−4^) for two hours at 37°C and 5% CO_2_. The cells were then washed three times with fresh media to remove excess virus. CD4 T cell targets isolated from PBMC (obtained from HIV-1 seronegative donors) were activated with IL2 and PHA as described above and were added into the DC culture at a DC to CD4 ratio of 1:10. After four days, cells were labeled with dead cell dye (Invitrogen), anti-CD3-Pacific Blue (BD Pharmingen), and anti-CD4-alexa 780 (BD Pharmingen) at 4°C for 30 min. The cells were permeabilized and labeled with anti-gag p24-PE (BD Pharmingen). Events were acquired on an LSR II flow cytometer and Gag-p24 expression was quantified in CD3^+^/CD4^+^ cells.

### Viral sequencing

Viral RNA was extracted from plasma using QIAamp RNA mini kits (Qiagen, Valencia, CA) and cDNA synthesis was carried out using Superscript IV (Invitrogen) [75]. cDNA synthesis was initiated by outer reverse primer: 5’- TAA CCC TGC GGG ATG TGG TAT TCC -3’ for segment 1 (HXB2 position 691–2348); cDNA synthesis was initiated by outer reverse primer: 5’- CCC CTA GTG GGA TGT GTA CTT CTG -3’ for segment 2 (HXB2 position 2042–5187); cDNA synthesis was initiated by outer reverse primer: 5’—GCA CTC AAG GCA AGC TTT ATT GAG GC -3’ for segment 3 (HXB2 position 4954–9557). The nested PCR reactions were carried out by using Q5 Hot Start High-Fidelity DNA Polymerase (NEB). The segment 1 first round PCR primers were: sense primer 623F+ 5’- AAA TCT CTA GCA GTG GCG CCC GAA CAG—3’; anti sense primer 2CRX- 5’- TAA CCC TGC GGG ATG TGG TAT TCC—3’; the second round primers were: sense primer G1+ 5’- GCA GGA CTC GGC TTG CTG AAG CGC—3’; anti sense primer G10- 5’- TAC TGT ATC ATC TGC TCC TGT ATC—3’. The segment 2 first round PCR primers were: sense primer P1+ 5’- GAA AAA GGG CTG TTG GAA ATG TGG—3’; anti sense primer P17- 5’- CCC CTA GTG GGA TGT GTA CTT CTG-3’; second round primers were: sense primer P2+ 5’- AGG AAG GAC ACC AAA TGA AAG-3’; anti sense primer P16- 5’- GGA TGA GTG CTT TTC ATA GTG A-3’. The segment 3 first round PCR primers were: sense primer FB6+ 5’- GCA TTC CCT ACA ATC CCC AAA G-3’; anti sense primer FB12- 5’- GCA CTC AAG GCA AGC TTT ATT GAG GC-3’; second round primers were: sense primer FB7+ 5’- TCT GGA AAG GTG AAG GGG CAG TAG-3’; anti sense primer FB13- 5’- GGT CTA ACC AGA GAG ACC CAG TAC AG-3’ [76]. PCR products were electrophoresed on an agarose gel to confirm the presence of the target DNA and further purified by Qiaquick PCR purification kit (Qiagen). The PCR products are sequenced by making 6 SMRTbellTM barcoded libraries which contains multiple HIV-1 PCR amplicons from multiple patients (PacBio Template prep kit). Each library was constructed by pulling same segment multiple PCR amplicons from multiple patients in equimolar amounts and based on the length of the amplicons. Libraries were sent to University of Delaware DNA Sequencing & Genotyping Center for PacBio sequencing. Sequence data was derived from MDPseq work flow. Sequences were analyzed phylogenetically using Geneious software (Biomatters, Auckland, NZ).

### Statistical analysis

Data were analyzed using Fisher’s exact t test; Mann-Whitney test for unpaired comparison; nonparametric Wilcoxon ranked test (two-tailed) for paired comparison and Pearson correlation analysis. Specifically, for the data in Figs 1B and 8B, we applied a mixed effects model to account for structural variability of our data. GraphPad Prism (version 7.0) was used to perform these analyses. Significance was determined as p value < 0.05.

## Supporting information

S1 Fig Gating strategies for flow based functional assays. The gating strategies for cytotoxicity assay **(A)**, ICS/phenotyping assay **(B)**, DC maturation assay **(C)**, and viral *trans*-infection assay **(D)** are shown. The cell population gated in each figure is defined at the top of the figure.
(TIF)

S2 Fig Similar functionality of NAE and AE-CD8s as assessed in an *ex vivo* ELISpot and an intracellular cytokine staining assay. **(A)** Magnitude of CD8 T cell IFNγ response measured in 7 dual positive PBMCs responding to 7 NAE and AE pairs in cytotoxicity assay is shown **(B-E)** The effector/cytokine production and **(F)** polyfunctionality of epitope specific CD8 T cells lines is shown. Wilcoxon matched-pairs signed rank test were used to determine statistical significance.
(TIF)

S3 Fig Gag-SL9 variant epitopes can promote DC maturation and HIV *trans*-infection. **(A)** Gag-SL9 clone generated from an HLA-A*02 expressing HIV naïve individual was tested in an IFNγ ELISpot assay for responses to primary peptide SL9 (SLYNTVATL, NAE) as well as its cross-reactive variants SFL9 (SLFNTVATL, AE) and SVL9 (SLYNTVAVL, AE). Magnitude of response to the three aforementioned peptides is shown. **(B)** Monocytes were isolated and treated with GM-CSF and IL4 to generate immature DCs (iDCs), which were then cultured with or without the Gag-SL9 clone pulsed with SL9, SFL9 or SVL9 peptide. Impact of each peptide-pulsed Gag-SL9 clone on the maturation status of iDCs was determined by surface expressions of CD83 and CD86. iDC cultured in maturation cocktail (as described in Methods) was used as positive control while iDC culture without treatment was used as negative control. **(C)** Activated CD4 T cells from the same HIV naïve donor were cultured in the presence of TFV based R5 tropic virus (*cis*-infection) at two different MOIs (10^−4^ or 10^−1^). CD4 T cells cultured without virus was used as a negative control. Percentage of infected target cells as shown by Gag p24 expression is indicated. **(D)** iDCs were cultured with NAE or AE stimulated CD8 T cell lines. After removal of CD8 T cells, DCs were then loaded with HIV-1 virus (MOI = 10^−4^) and co-cultured with activated CD4 T cells (*trans*-infection) isolated from an HIV naïve donor. In both (**C)** and (**D)**, viral infectivity was quantified by HIV Gag p24 expression within CD4 T cells (both CD4^hi^ and CD4^lo^ T cell populations) using flow cytometry. **(E)** Cumulative data of viral *cis-* and *trans-*infections at different MOIs obtained from 4 individuals is shown. To determine statistical significance, Mann–Whitney U test was used in **(E)**.
(TIF)

S4 Fig Similar CD8 IFNγ responses (NAE or AE) under *ex vivo* conditions. Magnitude of CD8 T cell IFNγ response measured in 16 dual positive PBMCs responding to 26 NAE and AE pairs in antigen sensitivity assays is shown.
(TIF)

S5 Fig Representative data from a single individual (CHI-1) responding to Env-B*0702-IL9/IF9 in each of the functional assays used in this study. The representative examples from CHI-1 for cytotoxicity assay **(A)**, DC maturation assay **(B)**, viral *trans*-infection assay **(C)**, and antigen sensitivity assay **(D)** are shown.
(TIF)

S1 Table Predicted non-adapted epitopes (NAE) and their corresponding adapted epitope (AE). (PDF)

S2 Table The response frequency to unique NAE/AE measured during acute and chronic HIV infection (Longitudinal cohort, N = 13). (PDF)

S3 Table Frequency of 7AAD+ CD4 T cells measured in an *in-vitro* cytotoxicity assay using PBMC samples obtained from chronically HIV infected (CHI) individuals. (PDF)
