# Supplementary figures and images for: CD8 T cells targeting adapted epitopes in chronic HIV infection promote dendritic cell maturation and CD4 T cell trans-infection
Source: PLoS Pathog. 2019 Aug 9;15(8):e1007970. doi: 10.1371/journal.ppat.1007970 (PMC6703693; doi:10.1371/journal.ppat.1007970)

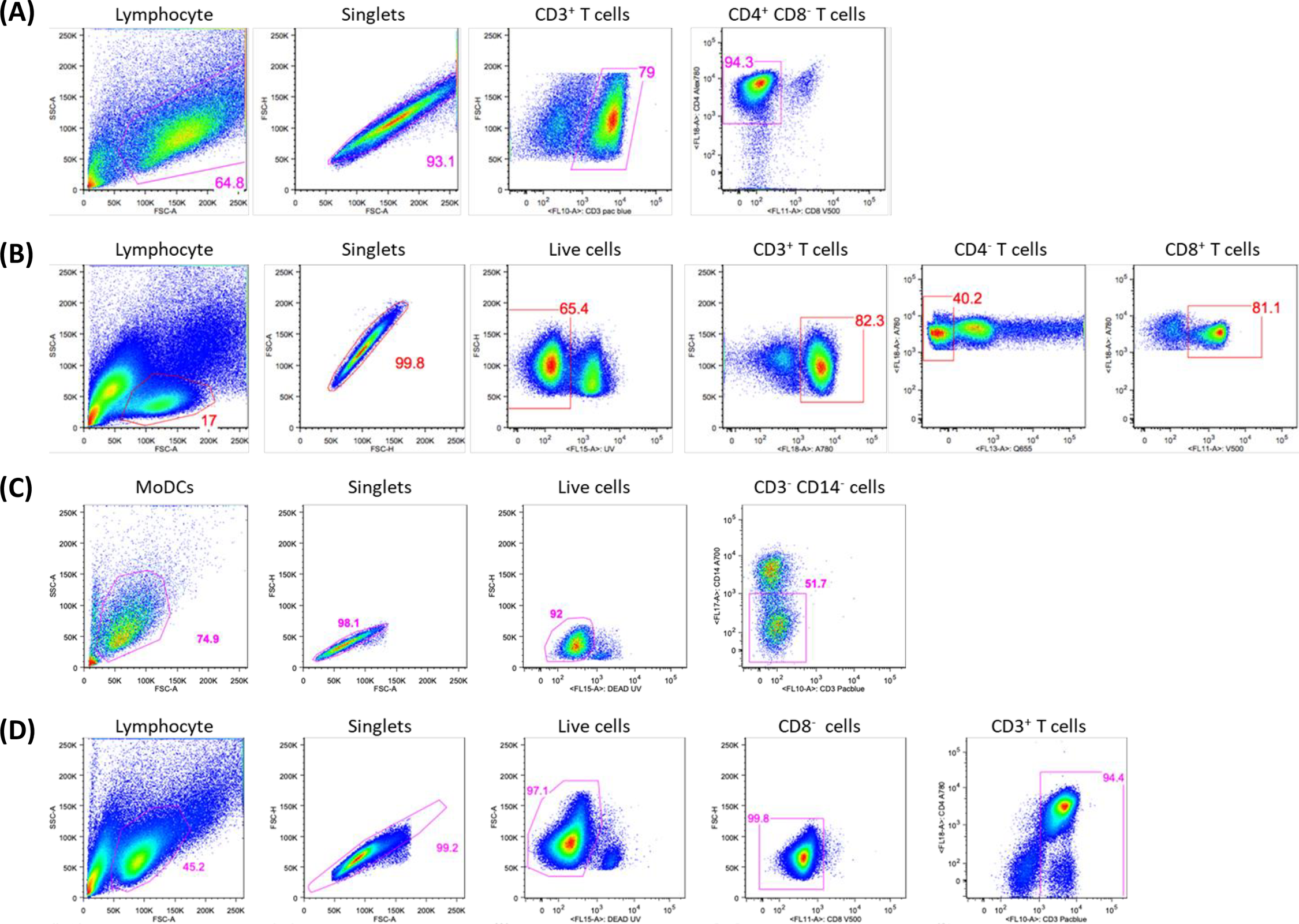

Supplement: S1 Fig — The gating strategies for cytotoxicity assay (A), ICS/phenotyping assay (B), DC maturation assay (C), and viral trans-infection assay (D) are shown. The cell population gated in each figure is defined at the top of the figure. (TIF) [file ppat.1007970.s001.tif]

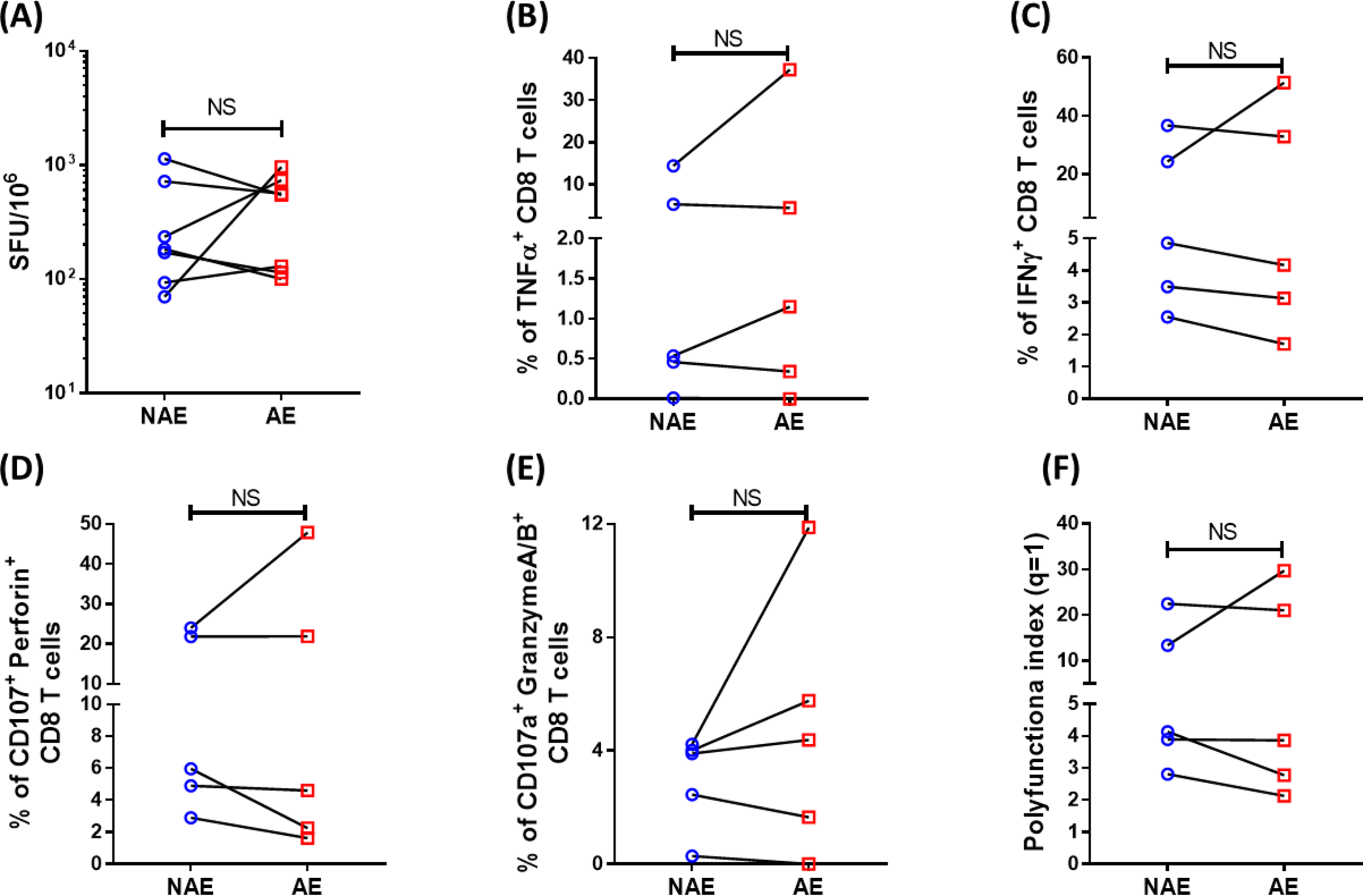

Supplement: S2 Fig — (A) Magnitude of CD8 T cell IFNγ response measured in 7 dual positive PBMCs responding to 7 NAE and AE pairs in cytotoxicity assay is shown (B-E) The effector/cytokine production and (F) polyfunctionality of epitope specific CD8 T cells lines is shown. Wilcoxon matched-pairs signed rank test were used to determine statistical significance. (TIF) [file ppat.1007970.s002.tif]

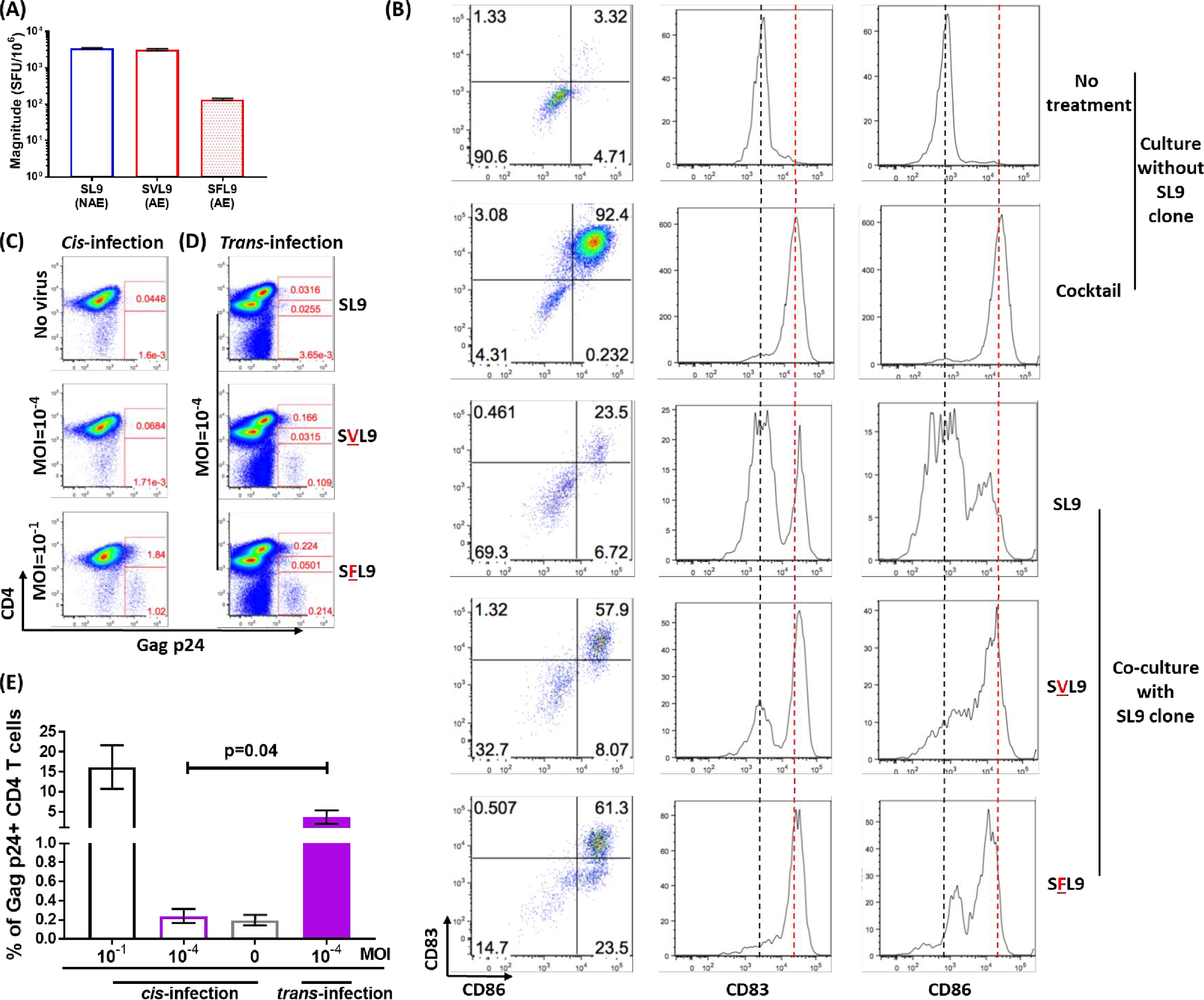

Supplement: S3 Fig — (A) Gag-SL9 clone generated from an HLA-A*02 expressing HIV naïve individual was tested in an IFNγ ELISpot assay for responses to primary peptide SL9 (SLYNTVATL, NAE) as well as its cross-reactive variants SFL9 (SLFNTVATL, AE) and SVL9 (SLYNTVAVL, AE). Magnitude of response to the three aforementioned peptides is shown. (B) Monocytes were isolated and treated with GM-CSF and IL4 to generate immature DCs (iDCs), which were then cultured with or without the Gag-SL9 clone pulsed with SL9, SFL9 or SVL9 peptide. Impact of each peptide-pulsed Gag-SL9 clone on the maturation status of iDCs was determined by surface expressions of CD83 and CD86. iDC cultured in maturation cocktail (as described in Methods) was used as positive control while iDC culture without treatment was used as negative control. (C) Activated CD4 T cells from the same HIV naïve donor were cultured in the presence of TFV based R5 tropic virus (cis-infection) at two different MOIs (10−4 or 10−1). CD4 T cells cultured without virus was used as a negative control. Percentage of infected target cells as shown by Gag p24 expression is indicated. (D) iDCs were cultured with NAE or AE stimulated CD8 T cell lines. After removal of CD8 T cells, DCs were then loaded with HIV-1 virus (MOI = 10−4) and co-cultured with activated CD4 T cells (trans-infection) isolated from an HIV naïve donor. In both (C) and (D), viral infectivity was quantified by HIV Gag p24 expression within CD4 T cells (both CD4hi and CD4lo T cell populations) using flow cytometry. (E) Cumulative data of viral cis- and trans-infections at different MOIs obtained from 4 individuals is shown. To determine statistical significance, Mann–Whitney U test was used in (E). (TIF) [file ppat.1007970.s003.tif]

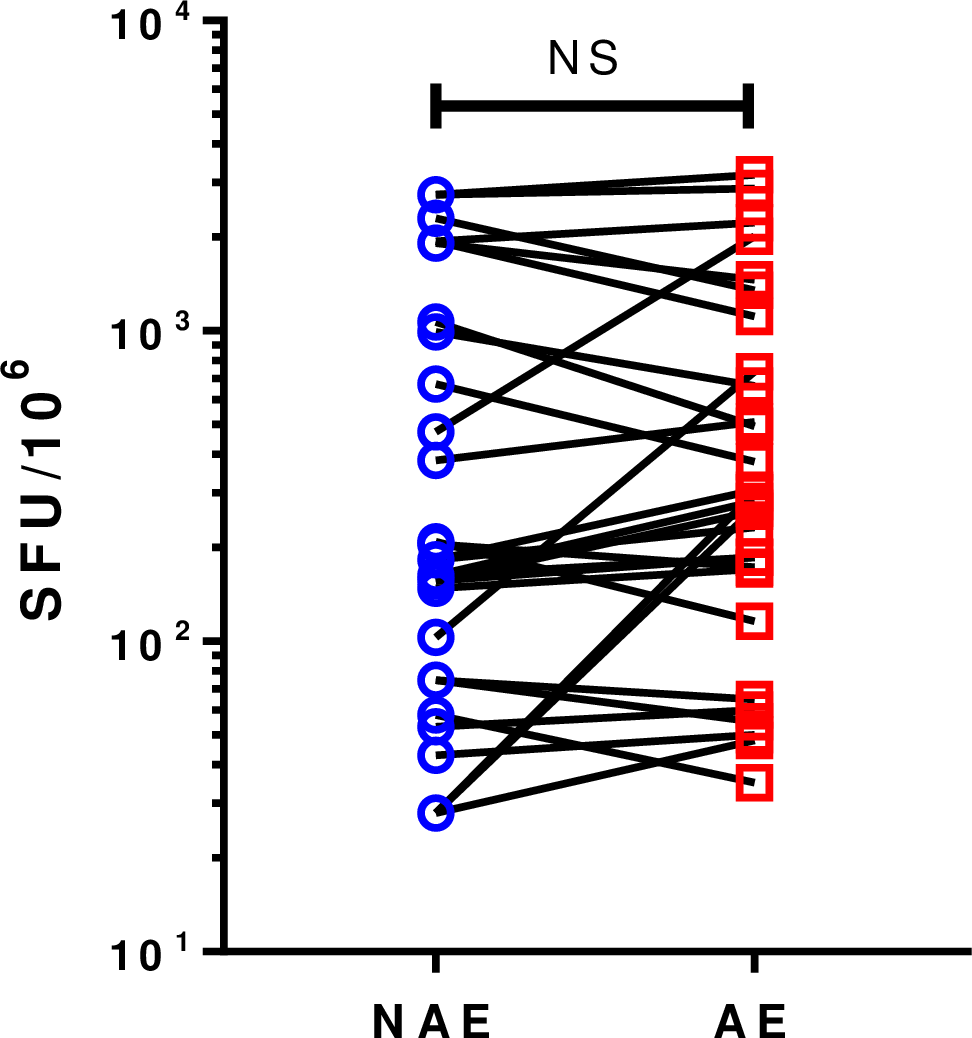

Supplement: S4 Fig — Magnitude of CD8 T cell IFNγ response measured in 16 dual positive PBMCs responding to 26 NAE and AE pairs in antigen sensitivity assays is shown. (TIF) [file ppat.1007970.s004.tif]

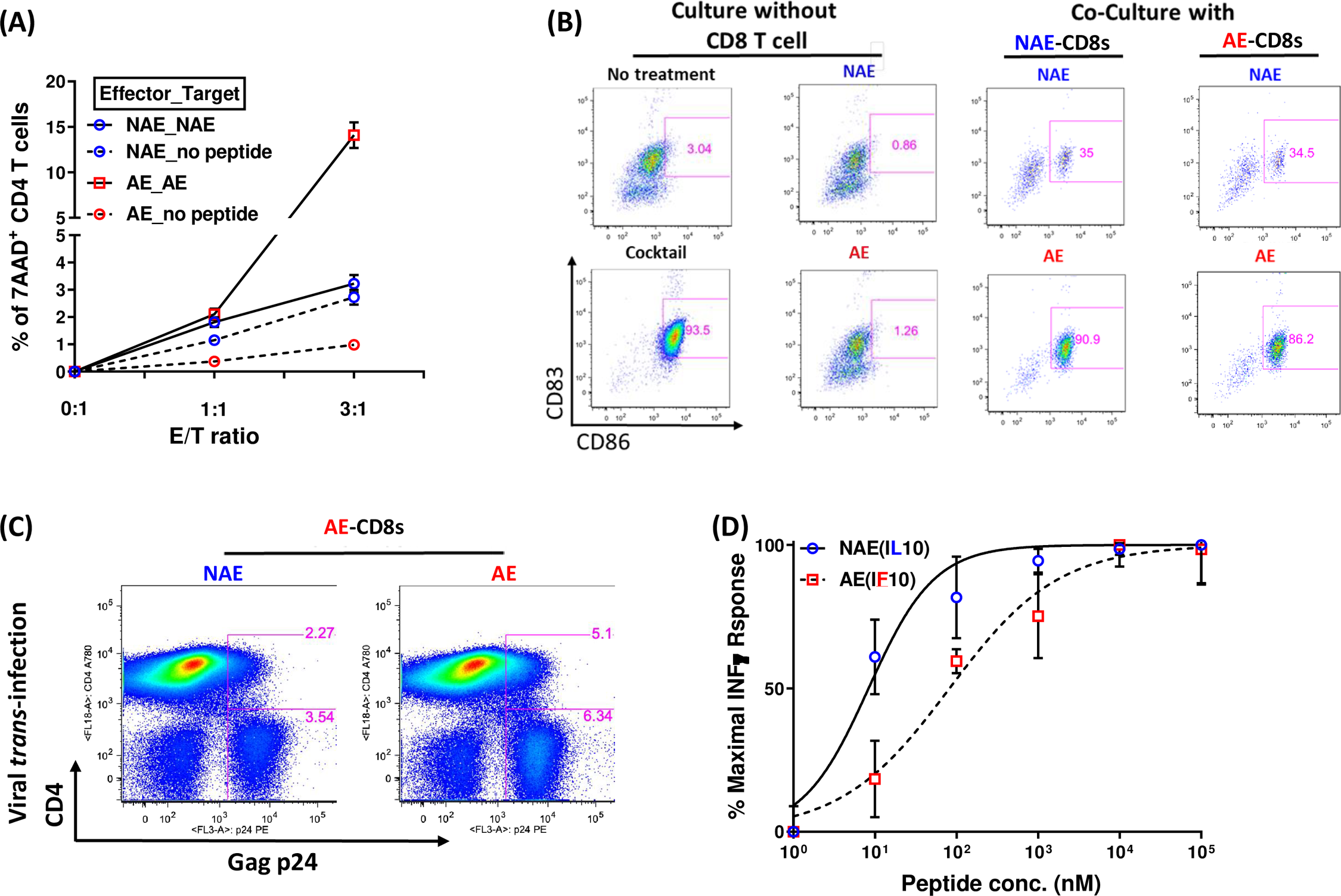

Supplement: S5 Fig — The representative examples from CHI-1 for cytotoxicity assay (A), DC maturation assay (B), viral trans-infection assay (C), and antigen sensitivity assay (D) are shown. (TIF) [file ppat.1007970.s005.tif]
