# Supplementary material for: CD8 T cells targeting adapted epitopes in chronic HIV infection promote dendritic cell maturation and CD4 T cell trans-infection
Source: PLoS Pathog. 2019 Aug 9;15(8):e1007970. doi: 10.1371/journal.ppat.1007970 (PMC6703693; doi:10.1371/journal.ppat.1007970)
Supplement: S1 Table — (PDF) [file ppat.1007970.s006.pdf]

Supplementary Table 1. Predicted non-adapted epitopes (NAE) and their corresponding adapted epitope (AE).

HLA-I Restriction	Protein	NAE sequence	Binding Affinity[a]	AE sequence[b]	Binding Affinity	Anchor Mutation[c]
A*02:01	Env	SLLNATAIAV	20.3	SLFNATAIAV	14.5	No
A*03:01	Env	TVYYGVPVWK	25.2	TIYYGVPVWK	22.7	Yes
A*24	Env	NYTSLIYTL	26.8	NYTGLIYTL	43.7	No
B*07	Env	IPRRIRQGL	6	IPRRIRQGF	17.1	Yes
A*01	Gag	SLYNTVATLY	1207.8	SLFNTVATLY	1226	No
A*02	Gag	SLYNTVATL	165.7	SLFNTVATL	105.4	No
				SLYNTVVTL	64.7	No
A*03:01	Gag	KIRLRPGGK	89.6	RIRLRPGGK	77.6	No
A*03:01	Gag	RLRPGGKKK	88.6	**RLRPGGKKQ**	6658.5	Yes
				**RLRPGGKKR**	875.5	Yes
A*03:01	Gag	RLRPGGKKKYK	139.7	RLRPGGKKQYK	60.1	No
				RLRPGGKKRYK	101.6	No
A*30	Gag	RLRPGGKKKY	732.1	RLRPGGKKNY	557.1	No
				RLRPGGKKRY	1121.6	No
B*08:01	Gag	IYKRWIIL	1034.7	**IYKRWIII**	4131.6	Yes
A*11	Gag	ATLYCVHQK	9.2	AVLYCVHQK	15.2	Yes
A*11	Gag	QMVHQPISPR	1588.5	QMVHQAISPR	2343.5	No
A*11:01	Gag	TLYCVHQR	4959	TLYCVHQK	695.9	Yes
A*11:01	Gag	ACQGVGGPGHK	16255.2	ACQGVGGPSHK	11188.9	No
A*11:01	Gag	VTNSATIMMQK	47.1	**VTNSATIMMQR**	563.3	Yes
A*24	Gag	KYKLKHIVW	602.6	KYRLKHIVW	840	No
A*30	Gag	RLRPGGKKKY	732.1	RLRPGGKKRY	1121.6	No
				RLRPGGNKKY	296.6	No
B*14	Gag	DRWEKIRL	2040.1	DKWEKIRL	10460.5	Yes
B*07:02	Gag	GPSHKARVL	35.2	GPSHKARIL	71.6	No
B*35:01	Gag	NPPIPVGDIY	83.3	NPPIPVGEIY	107.6	No
B*40:01	Gag	IEVKDTKEAL	121.7	IDVKDTKEAL	16478.5	Yes
B*40:02	Gag	TERQANFL	27734.6	NERQANFL	36680.4	No
B*44	Gag	AEQASQDVKNW	17.3	AEQASQEVKNW	17.2	No
B*52:01	Gag	RMYSPTSILDI	5331.9	RMYSPSSILDI	5258.5	No
B*57	Gag	TSTLQEQIGW	72.9	TSNLQEQIGW	82.2	No
				TSTLQEQIAW	69.9	No
B*58	Gag	TTSTLQEQIGW	74.1	TTSNLQEQIGW	93.5	No
				TTSTLQEQIAW	59.5	No
				TTSTLQEQVGW	74.8	No
C*06:02	Gag	YCVHQRIEV	22489.2	YCVHQKIEV	26641	No
A*02	Nef	FLKDKGGLEGL	469.6	FLKEKGGLEGL	333.2	No
A*03:01	Nef	AVDLSHFLK	119.2	ALDLSHFLK	86.3	Yes
				**AVDLSHFLR**	1820.4	Yes

A*23:01	Nef	RYPLTFGWCF	7.1	RYPLTFGWCY	406	Yes
				RFPLTFGWCF	17.3	Yes
A*24	Nef	RYPLTFGWCF	8.1	RYPLTLGWCF	11.9	No
				RFPLTFGWCF	21.4	Yes
A*30:02	Nef	GYFPDWQNY	306.7	GFFPDWQNY	226.3	Yes
A*30:02	Nef	HMAREKHPEFY	645.5	HMAREKHPEYY	578.9	No
B*07	Nef	FPVRPQVPL	12.2	FPVKPQVPL	13.3	No
B*07:02	Nef	FPVRPQVPLR	375.8	FPVKPQVPLR	345.8	No
B*07:02	Nef	RPMTYKGAL	2.3	RPMTFKGAL	2.3	No
B*07	Nef	TPGPGIRYPL	23.8	TPGPGVRYPL	23.6	No
				TPGPGTRYPL	20.5	No
B*07:02	Nef	VPVDPEKVEEA	18385.1	VPVEPEKVEEA	18311.8	No
B*15:03	Nef	LMWKFDSRLAF	7.7	LVWKFDSRLAF	16.5	Yes
B*35:01	Nef	KPQVPLRPMTY	7040.1	RPQVPLRPMTY	5333.3	No
B*35:01	Nef	TPGPGIRY	4328.7	TPGPGTRY	6039.2	No
				TPGPGVRY	4475.8	No
B*35:01	Nef	VPLRPMTYKGA	30986.1	VPLRPMDYKGA	32646.1	No
				VPLRPMTFKGA	30880	No
B*40:01	Nef	KEKGGLEGL	133.3	KEKGGLDGL	162.5	No
				**KDKGGLEGL**	5724	Yes
				KEKGGLEGI	237	Yes
				KEKGGLEGM	253.5	Yes
B*44:03	Nef	KEKGGLEGLIY	3069	**KDKGGLEGLIY**	27192	Yes
				**KEKGGLEGLIH**	28427.3	Yes
B*53:01	Nef	DPEKEVLVW	157.4	DPEKEVLAW	183.5	No
				DSEKEVLVW	1009.9	Yes
				DTEKEVLVW	1025.1	Yes
C*03:02	Nef	SLLHPMSQHGM	21098.2	SLLHPMNQHGM	22871.5	No
C*07	Nef	DILDLWVY	39007.9	DILDLWIY	40270.4	No
				DILDLWVH	45249.4	Yes
				EILDLWIY	39765.1	No
C*07:02	Nef	KRQDILDLWVY	9331.5	KRQDILDLWIY	11038.6	No
				KRQEILDLWVY	11322.7	No
A*03	Pol	AIFQSSMTK	12.3	AIFQSSMTR	72.7	Yes
				AIFQCSMTK	10.6	No
A*03:01	Pol	QIYAGIKVK	66.1	QIYPGIKVK	81.8	No
				**QIYAGIKVR**	1203.5	Yes
A*30	Pol	ILKEPVHGAYY	9236.3	ILKEPVHGVYY	10177.1	No
A*30:02	Pol	GQGQWTYQIY	120.4	GQDQWTYQIY	152.4	No
				GYGQWTYQIY	184.5	Yes
				**GHGQWTYQIY**	307.5	Yes
A*30:02	Pol	KIQNFRVYY	26	**KIQNFRVYF**	371	Yes
				KIQKFRVYY	44.6	No
				**NIQNFRVYY**	234.1	No

A*33:03	Pol	ELKKIIGQVR	381.6	ELKKIIEQVR	371	No
A*33:03	Pol	FYVDGAANR	64.8	FYVDGAASR	69.2	No
B*07:02	Pol	SPAIFQSSM	8.3	SPAIFQCSM	22	No
B*15:03	Pol	FKRKGGIGGY	95.1	FKRRGGIGGY	101.6	No
B*15:03	Pol	GHKAIGTVL	502.4	GKKAIGTVL	195.1	Yes
B*15:03	Pol	LMWKFDSRLAF	7.7	LVWKFDSRLAF	16.5	Yes
B*15:03	Pol	RKAKIIRDY	31.9	RKVKIIRDY	47.4	No
B*15:10	Pol	ILKEPVHGVY	31171.8	ILREPVHGVY	27490.2	No
B*35:01	Pol	NPDIVIYQY	33.3	NPEIVIYQY	71.4	No
B*35:01	Pol	VPLDEDFRKY	343.8	VPLDEEFRKY	425.7	No
				VPLDKDFRKY	557.3	No
B*44	Pol	AEIQKQGQGQW	56.2	AEIQKQGDGQW	81.9	No
				AELQKQGQGQW	91	No
B*44:02	Pol	IDKAQEEHERY	31255.5	IDKAQEEHEKY	31359.8	No
B*44:03	Pol	EEMNLPGRW	15.2	EDMNLPGRW	485.7	Yes
B*51:01	Pol	TAFTIPSI	326.6	TAFTIPSV	549.4	Yes
B*58	Pol	ITTESIVIW	5.2	IATESIVIW	5.6	Yes
C*04	Pol	AYFILKLA	37046.5	AYFLLKLA	36258.6	No
A*30	Rev	KTVRLIKFLY	115.5	KTVRLIKILY	97.5	No
B*07:02	Rev	RPAEPVPLQL	26.4	RPTEPVPLQL	62.2	No
				**RSAEPVPLQL**	8118.9	Yes
B*53:01	Tat	EPVDPRLEPW	147.4	DPVDPRLEPW	227.3	No
B*07	Vif	HPRISSEVHI	59.7	**HPKISSEVHI**	540.4	No
A*02:01	Vpr	AIIRILQQL	450.4	ALIRILQQL	27.8	Yes
				AIIRMLQQL	732.2	No
B*07:02	Vpr	FPRPWLHSL	4.4	FPRPWLHGL	10.1	No
B*27	Vpr	SRIGITRQRR	26136.2	SRIGITRQTR	21719.2	No

[a] Peptide-HLA binding affinity (nM) predicted using NetMHCpan Server

[b]Adaptations impacting predicted HLA binding affinity are bolded, i.e. a change from strong binding to weak binding or from weak binding to no binding, certain NAE had more than one predicted AE; red = site of adaptation

[c] Mutations occuring at p2 or the C-terminal of epitope are defined as anchor mutations
