# Supplementary material for: CD8 T cells targeting adapted epitopes in chronic HIV infection promote dendritic cell maturation and CD4 T cell trans-infection
Source: PLoS Pathog. 2019 Aug 9;15(8):e1007970. doi: 10.1371/journal.ppat.1007970 (PMC6703693; doi:10.1371/journal.ppat.1007970)
Supplement: S2 Table — (PDF) [file ppat.1007970.s007.pdf]

Supplementary Table 2. The response frequency to unqiue NAE/AE measured during acute and chronic HIV infection (Longitudinal cohort, N=13)

Epitope Group	HLA-I restriction	Protein	Predicted epitope[a]	TFV encoding epitope[b]	Tested [c]	Response[d]	
						Acute	Chronic
AE	A*02	Env	SLFNATAIAV	NLFNTTAIAV	1	0	1
	A*24	Env	NYTGLIYTL	NYTGIIYTL	1	1	1
	B*44	Gag	AEQASQEVKNW	AEQASQEVKNW	3	2	3
	B*07:02	Gag	GPSHKARIL	GPGHKARIL	1	0	0
	B*35	Gag	NPPIPVGEIY	NPPIPVGEIY	1	0	0
	A*03:01	Gag	RLRPGGKKQ	RLRPGGKKQ	1	0	0
	A*01	Gag	SLFNTVATLY	SLFNTVATLY	2	0	0
	A*11:01	Gag	TLYCVHQK	TMYCVHQK	1	0	1
	A*03:01	Nef	ALDLSHFLK	ALDLSHFLK	2	1	0
	A*03:01	Nef	ALDLSHFLK	ALDLSHFLR	1	0	1
	C*7	Nef	DILDLWIY	DILDLWIY	1	0	0
	A*02	Nef	FLKEKGGLEGL	FLKEMGGLEGL	1	1	1
	A*02	Nef	FLKEKGGLEGL	FLKENGGLEGL	1	0	1
	B*07:02	Nef	FPVKPQVPL	FPVKPQVPL	1	1	1
	B*07:02	Nef	FPVKPQVPLR	FPVKPQVPLR	1	0	1
	A*30	Nef	HMAREKHPEYY	HVARELHPEYY	1	0	0
	B*44	Nef	KEKGGLEGLIH	KEMGGLEGLIH	1	0	1
	B*44	Nef	KEKGGLEGLIH	KENGGLEGLIH	1	0	1
	C*07:02	Nef	KRQEILDLWVY	KRQEILDLWVY[1]	1	0	1
	A*23:01	Nef	RFPLTFGWCF	RFPLTFGWCF	1	1	1
	B*07:02	Nef	RPMTFKGAL	RPMTFKGAL	1	1	0
	B*07:02	Nef	TPGPGTRYPL	TPGPGTRYPL	1	0	1
	B*07	Nef	VPVEPEKVEEA	VPMEPEKIEEA	1	0	0
	B*07	Nef	VPVEPEKVEEA	VPMEPEKVEEA	1	0	0
	B*44	Pol	AELQKQGQGQW	AELQKQGQGQW	1	1	1
	A*03:01	Pol	AIFQCSMTK	AIFQCSMTK	2	0	0
	C*04	Pol	AYFLLKLA	AYFLLKLA	2	0	1
	B*44:03	Pol	EDMNLPGRW	EDMNLPGRW	1	0	0
	B*44	Pol	IDKAQEEHEKY	IDKAQEDHEKY	1	0	1
	B*44:02	Pol	IDKAQEEHEKY	IDKAQEEHEKY	1	0	0
	A*30	Pol	ILKEPVHGVYY	ILKEPVHGVYY	1	0	0
	A*30	Pol	NIQKFRVYY	NLQKFRVYY	1	0	0
	A*03:01	Pol	QIYPGIKVK	QIYPGIKVK	1	1	1
	B*35	Pol	VPLDKDFRKY	VPLDKDFRKY	1	0	0
	A*02:01	Vpr	ALIRILQQL	ALLRSLQQL	1	0	1
	B*07	Vpr	FPRPWLHGL	FPRMWLHGL	1	0	1
	B*07:02	Vpr	FPRPWLHGL	FPRVWLHGL	1	0	0
	A*02	Env	SLLNATAIAV	NLLNTTAIAV	1	1	1
	A*02	Env	SLLNATAIAV	SLLNATAIAV	1	0	0
	A*03:01	Env	TVYYGVPVWK	TVYYGVPVWK	1	1	0

NAE	B*07	Env	IPRRIRQGL	VPVRIRQGL	1	0	0
	A*11:01	Gag	ACQGVGGPGHK	ACQGVGGPGHK	1	0	1
	B*44	Gag	AEIQKQGQGQW	AEIQKQGQGQW	1	1	0
	B*08:01	Gag	IYKRWIIL	IYKRWIIL	2	0	1
	A*03:01	Gag	KIRLRPGGK	KIRLRPGGK	2	0	0
	A*24:02	Gag	KYKLKHIVW	KYKLKHIVW	1	0	1
	A*03:01	Gag	RLRPGGKKK	RLRPGGKKK	2	0	2
	A*30	Gag	RLRPGGKKKY	RLRPGGKKKY	1	0	0
	A*03:01	Gag	RLRPGGKKKYK	RLRPGGKKKYR	1	0	1
	B*52:01	Gag	RMYSPTSILDI	RMYSPTSILDI	1	1	0
	A*01	Gag	SLYNTVATLY	SLYNTVATLY	1	0	0
	A*03:01	Nef	AVDLSHFLK	AVDLSHFLK	1	1	0
	C*07	Nef	KRQDILDLWVY	DILDLWVY	2	0	0
	B*07:02	Nef	FPVRPQVPL	FPVRPQVPL	1	1	1
	B*07:02	Nef	FPVRPQVPLR	FPVRPQVPLR	2	0	0
	A*30:02	Nef	GYFPDWQNY	GYFPDWQNY	1	0	0
	B*07:02	Nef	RPMTYKGAL	RPMTYKGAL	3	1	3
	A*23:01	Nef	RYPLTFGWCF	RYPLCFGWCF	1	1	1
	A*24:02	Nef	RYPLTFGWCF	RYPLTFGW	1	0	1
	C*03:02	Nef	SLLHPMSQHGM	TLLHPMSQHGM	1	0	0
	B*07:02	Nef	TPGPGIRYPL	TPGPGIRYPL	1	1	0
	B*35:01	Nef	VPLRPMTYKGA	VPLRPMTYKAA	1	1	1
	B*15:03	Pol	GHKAIGTVL	GHKAIGTVL	1	1	0
	B*15:10	Pol	ILKEPVHGVY	ILKEPVHGVY	1	1	0
	B*58:01	Pol	ITTESIVIW	ITTESIVIW	1	0	0
	A*03:01	Pol	QIYAGIKVK	QIYAGIKVK	1	1	1
	B*15:03	Pol	RKAKIIRDY	RKAKIIRDY	1	1	1
	B*07:02	Pol	SPAIFQSSM	SPAIFQSSM	1	0	1
	B*51:01	Pol	TAFTIPSI	TAFTIPSI	1	1	0
	A*30	Rev	KTVRLIKFLY	KTVRLIKRLY	1	0	0
	B*07	Rev	RPAEPVPLQL	RFEEPVPLQL	1	0	0
	B*07:02	Rev	RPAEPVPLQL	RPAEPVPLQL	2	1	1
	B*07	Vif	HPRISSEVHI	HPRISSQVYI	1	0	1

[a] Predicted NAE or AE. The polymorphic site is underlined and the adaptation at that site is shown in red/underlined

[b] NAE or AE encoded in TFV sequence for each patient

[c] Number of patients tested for the corresponding epitope at both stages of infection

[d] Number of patients responding to the corresponding epitope at each stage of infection
