# Supplementary material for: CD8 T cells targeting adapted epitopes in chronic HIV infection promote dendritic cell maturation and CD4 T cell trans-infection
Source: PLoS Pathog. 2019 Aug 9;15(8):e1007970. doi: 10.1371/journal.ppat.1007970 (PMC6703693; doi:10.1371/journal.ppat.1007970)
Supplement: S3 Table — (PDF) [file ppat.1007970.s008.pdf]

Supplementary Table 3. Frequency of 7AAD+ CD4 T cells measured in an in-vitro cytotoxicity assay using PBMC samples obtained from chronically HIV infected (CHI) individuals.

CMI	HLA restriction	Protein	Epitope		Normalized Frequency of 7AAD^{+} CD4 T cell [a]					
			NAE	AE	NAE			AE		
					0:1	1:1	3:1	0:1	1:1	3:1
CHI-1	B*07:02	Env	IPRRIRQGL	IPRRIRQG<u>F</u>	0.00	0.66	0.50	0.00	1.73	13.11
CHI-2	B*07:02	Nef	FPVRPQVPL	FPV<u>K</u>PQVPL	0.00	0.29	0.00	0.00	0.59	0.16
CHI-3	A*03:01	Gag	RLRPGGKKKYK	RLRPGGKK<u>Q</u>YK	0.00	0.00	1.11	0.00	0.32	4.60
CHI-4	B*15:03	Pol	FKRKGGIGGY	FKR<u>R</u>GGIGGY	0.00	14.45	45.30	0.00	42.88	58.48
CHI-5	B*35:01	Nef	TPGPGIRY	TPGPG<u>V</u>RY	0.00	0.00	0.06	0.00	0.00	0.23
CHI-6	B*44:02	Pol	AEIQKQGQGQW	AE<u>L</u>QKQGQGQW	0.00	0.09	6.38	0.00	1.62	27.49
CHI-7	B*35:01	Nef	TPGPGIRY	TPGPG<u>V</u>RY	0.00	0.69	8.54	0.00	1.38	21.63

[a] For response to each epitope, normalized data is shown for three different effector: target (E:T) ratios. Mean of experimental duplicates for each E:T ratio was first calculated and substracted from the corresponding negative control at the same E:T ratio. Finally, each data was normalized with respect to target only condition(E:T=0:1) set as 0.
